# Meat, milk, wool and grain: The animal economy of medieval England (CE 400 to 1400)

**DOI:** 10.1371/journal.pone.0355965

**Published:** 2026-09-02

**Authors:** Matilda Holmes, David Orton, Helena Hamerow, Richard Thomas

**Affiliations:** 1 School of Heritage and Culture, University of Leicester, Leicester, United Kingdom; 2 Department of Archaeology, University of York, York, United Kingdom; 3 Institute of Archaeology, Oxford University, Oxford, United Kingdom; University of Passau: Universitat Passau, GERMANY

## Abstract

Much has been written about the animal economy of medieval England (CE 450–1500) but interpretation of the zooarchaeological record has been held back by reliance on artificial periodisation. This study reduces that reliance via a novel approach to data synthesis. A substantial zooarchaeological dataset compiled for the ‘Feeding Anglo-Saxon England’ project was investigated allowing a more detailed understanding of when change took place and in what form. Key patterns include: a continuation of Roman husbandry practices at some sites throughout the fifth century; an increase in wool production from the early seventh century; the use of dedicated draught cattle from the ninth century, peaking in the eleventh and mid-twelfth centuries, after which the demand for milk, beef and increasing use of horses for arable production caused the use of cattle for draught to decline.

## Introduction

The medieval period in England (CE 450–1500) was a time of great change, which saw a re-emergence of urban life, changes in patterns of trade and the distribution of food, a growing population and increased social hierarchy from a largely subsistence-based economy (see for example [1–3]). The corresponding increase in food production required the development of animal husbandry to provide meat, fat and milk and facilitate the growth in arable output necessary to feed a population that had expanded from perhaps around one million in the post-Roman period to around 1.7–2.5 million in the eleventh century and six million by the 14^th^ century [4–6].

A large body of work already exists pertaining to the animal husbandry of medieval England. Zooarchaeological syntheses have hitherto been carried out on a regional basis [7–14], or on a broader scale but with a focus on limited chronological periods [15–24]. A significant limitation of previous syntheses has been the reliance on broad chronological periods, reflecting the conservatism of ceramic sequences, and the need to fit data into a small number of phases to facilitate comparison.

This paper provides a new perspective, utilising the largest dataset to date, to establish the animal economy on both a large geographic scale (most of England), and considerable temporal span (CE 400–1500). This work focuses on cattle and sheep as they had the greatest effect on the animal economy and utilises an approach to better understand fluctuations through time unhindered by inexact temporal labels such as ‘early Saxon’ and ‘high medieval’. Data come from the *FeedSax* project [25–27], which strived to provide a broader understanding of how agricultural change was implemented throughout England, including a more nuanced and wide-ranging consideration of underlying animal husbandry practices. This paper addresses a series of widely held hypotheses based on existing work to set the animal economy in context and refine the dating of transitions in food production. These are broadly summarised below and will be returned to in the course of the paper.

Hypothesis 1: During the period CE 450–650 there is a consensus that mixed farming was ubiquitous, with cattle and sheep used for small-scale dairy and wool production, plus traction, with no evidence for specialised or large-scale production [7,14,20,21,23,28].

Hypothesis 2: Cattle were important indicators of wealth and status in a fifth- to seventh-century society without a money economy, as indicated by their frequently observed predominance in assemblages [20–23,29,30].

Hypothesis 3: Greater diversity observed in relative proportions of the major domesticates between CE 650 and 850 was a response to a degree of specialisation driven by local decisions affecting husbandry regimes [14,19–21,29]. This resulted in the increasing use of sheep for wool and the provisioning of *wics* (trade centres) with beef, grain and raw materials.

Hypothesis 4: An increased emphasis on wool production from the ninth century has been inferred, often on a regional basis, continuing throughout the medieval period. This has been used to explain an increase in the proportions of sheep compared to other livestock, and their being kept longer [7,9,11,23].

Hypothesis 5: Changes in the animal economy in the wake of the Black Death (1348−9) included a move away from grain production towards an emphasis on wool, meat and milk [31].

Hypothesis 6: From the 15^th^ century there was a growing demand for meat and dairy products, inferred by the presence of a greater proportion of veal calves [7,9,19,24].

### A phase-independent approach to data analysis

Integrating quantitative data from multiple sites and phases with overlapping date ranges and varying resolution is a non-trivial challenge. Past approaches include: (a) assigning sites to pre-defined periods based on the mid-points of their date-ranges (e.g., [9]); (b) grouping phases together into periods – potentially overlapping – based on natural breaks in the data (e.g., [32]); (c) using Monte Carlo simulations to assign randomised calendar dates to assemblages (e.g., [33–35]); and (d) aoristic analysis, in which data from each phase are distributed across a number of chronological bins in proportion to the extent of their overlap with that assemblage’s reported date range (e.g., [33,36]) and on the assumption of equal probability across that range (although see [37]).

Methods based around grouping sites into periods benefit from simplicity but create a tension between resolution and accuracy: the broader the periods used, the greater the chance that the true date of an assemblage falls wholly or partly outside the period to which it is assigned (see discussion in [35]). This can be avoided by removing phases with long date ranges and/or those that substantially straddle period boundaries, but only at the expense of removing potentially important data from consideration and – worse – risking an artificial sharpening of differences between periods that may in fact represent gradual trends.

Monte Carlo methods have the advantage of capturing and representing the uncertainty inherent in archaeological dating but are most appropriate where reported date ranges largely represent uncertainty regarding the true date of deposition, e.g., when dealing with individual finds or features. Where reported date ranges represent a complex and indeterminate combination of the duration of activities and uncertainty over their timing, typically the case with zooarchaeological assemblages from entire phases as analysed here, aoristic analysis may be more appropriate [35]. Both these approaches maximise the number of assemblages that can be included while inherently reducing the weighting of more loosely dated assemblages at any given point within their reported date ranges. The latter point is crucial when the aim is to evaluate (for example) frequencies of rare taxa, isolated finds of which must date to discrete points within an assemblage’s reported data range. It is more questionable when looking at average frequencies of major taxa, however, since their remains are likely to have accumulated over much or all of the reported date range, and it is not clear that assemblages from short-lived sites should be weighted more heavily than those from major, long-lived settlements: once again there is a question of uncertainty versus duration.

Here, we compare two novel methods for tracking change over time in the proportions of major taxa, in this case cattle and sheep. First, we develop an aoristic mean. For each taxon in each assemblage, both its proportional contribution (e.g., 0.6 for 60% cattle) and an assemblage weight of 1 are apportioned across the years spanned by the phase’s date range. The aoristically weighted mean contribution of the taxon for a given year is then calculated as the sum of these apportioned proportions divided by the sum of the apportioned weights, such that assemblages with longer or less certain date ranges contribute proportionally less to the result for any single year. For example, a phase spanning 10 years with a cattle proportion of 0.6 would contribute 0.006 and a weight of 0.01 to each year, exerting less influence on the aoristic mean. Second, we apply a conceptually very simple approach based on *unweighted* mean, median, and percentiles. While novel, this method has been utilised for the *FeedSax* project where summary results were included [25], this paper provides a deeper investigation into the medieval animal economy using an expanded dataset.

The overarching aim of this paper is to refine our understanding of the animal economy of medieval England, to better realise how animals were used, and the changing focus of their exploitation. This has been achieved using an aoristic approach, allowing an analysis of continuous data between CE 400–1400.

## Methods

### The data set

Zooarchaeological data were incorporated from existing regional reviews from southern and central England [38–40], a survey of reports from northern counties, and those from previously reviewed areas published since 2008 to bring the dataset up to date. The data are available on the Archaeological Data Service [26]. Following the end date of the *FeedSax* project some additional sites were incorporated to allow better resolution of mortality data and the beginning and end ranges (Table 1). Sites were included if the reported occupation dates overlapped with the period CE 400–1400, produced a minimum of 100 total fragments of cattle, sheep and pig bones and had a date range up to 300 years. These were arbitrary cut off points, to provide the largest number of assemblages with a representative sample size, and to reduce uninformative ‘background noise’ from poorly dated material.

**Table 1:** Details of the supplementary data added after the end of the Feeding Medieval England project and therefore not included in the database.

Site	County	Site type
49-53 Commercial Road, Hereford [41]	Hereford and Worcester	Urban
Barrow Hills, Radley [42]	Oxfordshire	Rural
Bayley Lane, Coventry (Holmes unpublished)	Warwickshire	Urban
Botolph Bridge, Orton Longueville [43]	Cambridgeshire	Manor
Broad Street, Reading [44]	Berkshire	Urban
East Challow [45]	Oxfordshire	Rural
Hornsea [46]	Lincolnshire	Rural
Mount Farm, Berinsfield [47]	Oxfordshire	Rural
Pathfinder House, Huntingdon [48]	Cambridgeshire	Urban
Redcliffe [49]	Somerset	Urban
The Still, Peterborough [50]	Cambridgeshire	Urban
Wigmore Castle [51]	Hereford and Worcester	High-Status
Winchester 2002–2007 [52]	Hampshire	Urban

The focus of this research is on cattle and sheep. Although pigs make a vital contribution to the non-agrarian economy of medieval England, their primary importance for meat and limited contribution to the wider animal economy beyond manure, means that they are excluded from this study, although several excellent investigations into pig husbandry exist (for example [53–56]). The bones of sheep and goat are morphologically similar and are commonly grouped together in specialist reports, with inconsistency in species-level identification rates necessitating their grouping in synthetic studies [57], but in the present context the overwhelming majority belong to sheep [58,59] and so it is this taxon that will be referred to throughout.

For clarification, a ‘site’ is the name given to each excavation, ‘assemblage’ refers to the animal remains produced from a single phase within that excavation, and ‘phase’ is the discrete date-range assigned in the site report. Thus, a single site may have more than one assemblage, each allocated to a specific phase. Maps were created by the authors in QGIS [60] using baseline data provided by Lowerre *et al.* [61] based on the work of Roberts and Wrathmell [62] and Rippon et al. [63]. Data analysis was carried out in R [64] using the *data.table* package [65], and the code is available from the Center for Open Science repository (https://osf.io/kfsrz/overview?view_only=1e1611fd99974b258060ea14117ff77d)

### Distribution of sites

**Fig 1:**
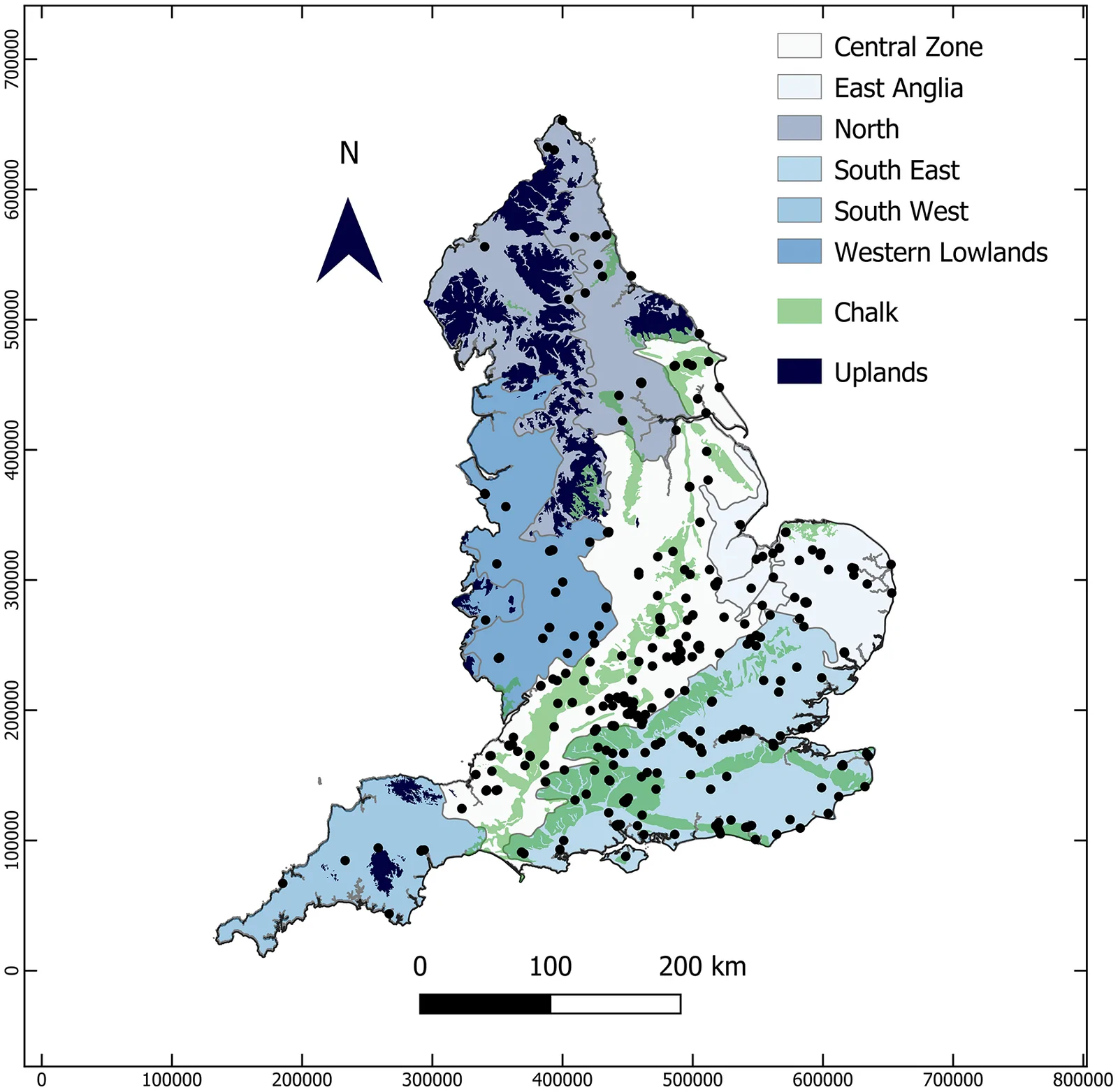
Distribution of all sites included in the data set. Showing the zones referred to in the text [63], chalk and upland areas over 300m/ 1000 ft [61]. Original map created by M Holmes using QGIS (http://www.qgis.org, accessed 20/11/2025).

701 assemblages from 460 sites were included. Fig 1 shows the distribution of all sites in the database, and Table 2 summarises the data by date range, region, geology and site type. Sites were placed into broad regional groups defined by Rippon *et al.* in the *Fields of Britannia* project [63]. The Central Zone and South East are best represented in the dataset, which is not unexpected as these regions cover the greatest landmass (Fig 1). The Northern region was originally divided into the Northern Uplands and North East Lowlands [63], but none of the sites in this study are in the uplands (i.e., areas over 300m above ordnance datum) and very few come from the Northern Lowland region, so the two regions were combined. Similarly, only a maximum of three assemblages contribute to the Wash and Fens region for any single date range, so this has been amalgamated with East Anglia. Areas of poor coverage (notably the North West, South West and parts of the South East of the country) correspond to acidic soils, which are not conducive to bone preservation [66]. Settlements on peaty or sandy soils will therefore be under-represented in the zooarchaeological record.

**Table 2:** Number of assemblages within each site type, region and geology. 25 year date ranges replace artificial phasing, allowing aoristic changes in the data to be identified.

Years CE	Site type					Region						Geology				
	Rural	Urban	Ecclesiastical	High-Status	Total	Central Zone	East Anglia	North	South East	South West	Western Lowlands	Chalk	Clay	Sand	Valley Terraces	Other
400-425	32			2	34	14	5	3	9	1	2	5	9	2	16	2
425-450	32			2	34	14	5	3	9	1	2	5	9	2	16	2
450-475	43	2		3	48	22	5	3	13	1	4	7	11	3	23	4
475-500	43	2		3	48	22	5	3	13	1	4	7	11	3	23	4
500-525	52	3		3	58	27	7	3	15	1	5	8	12	3	30	5
525-550	52	3		3	58	27	7	3	15	1	5	8	12	3	30	5
550-575	53	3		3	59	28	8	3	15	1	4	10	12	2	31	4
575-600	52	3		3	58	27	8	3	15	1	4	10	12	2	30	4
600-652	46	4	1	8	59	24	7	4	21	1	2	8	14	3	29	5
625-650	47	6	5	8	66	26	7	7	23	1	2	8	14	5	34	5
650-675	45	19	6	15	85	28	16	7	31	1	2	7	15	6	51	6
675-700	44	19	6	14	83	27	16	6	31	1	2	7	15	6	49	6
700-725	38	26	6	13	83	23	16	6	37		1	4	15	5	55	4
725-750	39	29	6	13	87	24	16	6	40		1	4	17	5	57	4
750-775	38	29	6	13	86	25	15	6	39		1	4	17	5	57	3
775-800	37	28	6	13	84	24	15	6	38		1	4	16	5	56	3
800-825	31	34	5	13	83	21	16	8	36	1	1	7	13	4	57	2
825-850	32	39	5	15	91	22	18	9	40	1	1	10	14	4	61	2
850-875	23	63	5	17	108	34	17	11	39	1	6	14	12	5	75	2
875-900	22	61	5	15	103	33	15	11	37	1	6	14	12	5	70	2
900-925	20	80	6	12	118	46	18	8	35	1	10	21	15	4	74	4
925-950	20	84	7	12	123	48	18	10	36	1	10	22	16	4	77	4
950-975	21	86	6	13	126	50	20	8	37	1	10	23	18	4	76	5
975-1000	21	85	6	13	125	50	20	8	36	1	10	22	18	4	76	5
1000-1025	27	119	8	23	177	69	27	9	49	7	16	28	33	6	104	6
1025-1050	29	128	8	24	189	73	26	11	55	7	17	31	37	6	107	8
1050-1075	33	146	12	34	225	90	27	12	70	8	18	39	42	6	127	11
1075-1100	28	114	10	30	182	74	21	9	55	8	15	34	38	5	96	9
1100-1125	33	123	9	32	197	74	15	14	64	12	18	32	36	3	116	10
1125-1150	33	128	10	36	207	78	15	16	67	13	18	34	38	3	122	10
1150-1175	42	142	14	43	241	96	21	14	79	11	20	43	40	3	147	8
1175-1200	41	137	14	41	233	91	21	13	78	10	20	42	39	3	142	7
1200-1225	31	124	10	38	203	85	14	16	62	6	20	26	42	2	127	6
1225-1250	32	130	14	42	218	91	14	19	66	7	21	28	46	2	137	5
1250-1275	36	137	11	42	226	93	14	18	68	10	23	27	49	2	142	6
1275-1300	35	133	11	40	219	92	14	17	64	9	23	26	49	2	136	6
1300-1325	26	87	5	26	144	59	10	8	45	7	15	20	32	1	87	4
1325-1350	26	86	4	25	141	59	10	8	44	7	13	20	31	1	85	4
1350-1375	19	71	4	15	109	48	9	7	32	2	11	14	25	1	66	3
1375-1400	19	66	3	14	102	44	9	7	29	2	11	13	24	1	62	2

### Analysis of chronological trends

Dating information was taken from the site reports. Where absolute dates were not available for an assemblage, they were interpreted broadly. For example, sites described as ‘early 5^th^ century to late 6^th^ century’ would be defined as dating to between CE 400 and 599. A ‘mid-5th century’ date would be recorded as CE 425–475. More general phases such as ‘early Saxon’ or ‘medieval’ were assigned dates as indicated in Table 2.

### Method 1: aoristic mean

**Fig 2:**
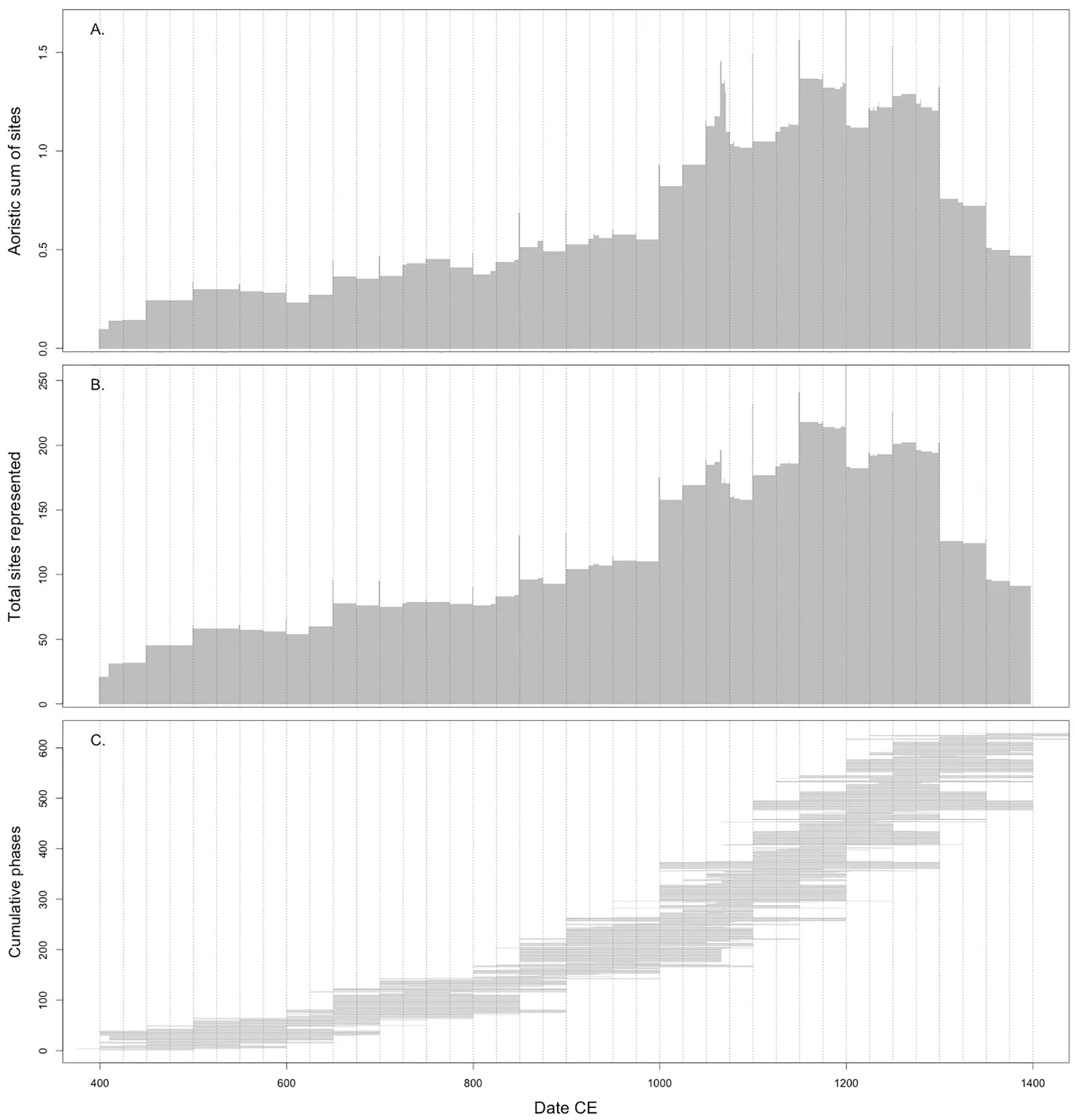
Chronological distribution of data included in study. A: aoristic sum of assemblages at one-year resolution; B: unweighted total of site phases overlapping with each year; C: date range of each individual assemblage, ordered by mid-point.

To calculate the aoristic mean, each assemblage was first assigned an equal total value of 1, which was then divided out across the years with which it overlaps – such that an assemblage spanning 100 years would contribute 0.01 to each. Each year falling within a given assemblage’s date range was thus assigned the reciprocal of the length of that date range. Totalling these values across all assemblages for each year gives the aoristic sum of assemblages contributing to the study (Fig 2A). This is clearly very similar to the graph of total number of sites contributing to the data at each year (Fig 2B), but with differences created by varying chronological resolution: assemblages with longer date ranges are spread out across more years and hence contribute less to the total weight in any given year. The actual chronological resolution of the assemblages contributing to these distributions can be better assessed via a radiator plot (Fig 2C).

Next, the proportion of cattle and sheep within each assemblage was calculated and the same procedure repeated as for the number of assemblages. For example, if cattle contribute 40% of an assemblage dated to 600–699 CE, a value of 0.004 (0.4/ 100) would be assigned to each year within that range. Again, these values were then totalled across all assemblages for each year, and the resulting aoristic sum for that species was divided by the aoristic sum of sites at each year. The outcome is a mean value for the contribution of a given species at each year that is weighted according to the chronological resolution of the contributing assemblages but not by their overall size.

### Method 2: unweighted means and quantiles

For the alternative approach, we simply calculated the mean, median, and quartiles of the frequency of a given taxon across all assemblages represented at each individual year in turn. All assemblages were thus equally weighted for any given year, but since longer-lived and/or more loosely dated assemblages contribute to a greater number of these annual snapshots they will have contributed more to the overall analysis than shorter-lived/more tightly dated assemblages. To the extent that they are longer-lived this is reasonable; where the longer date range largely represents uncertainty this is more problematic.

**Fig 3:**
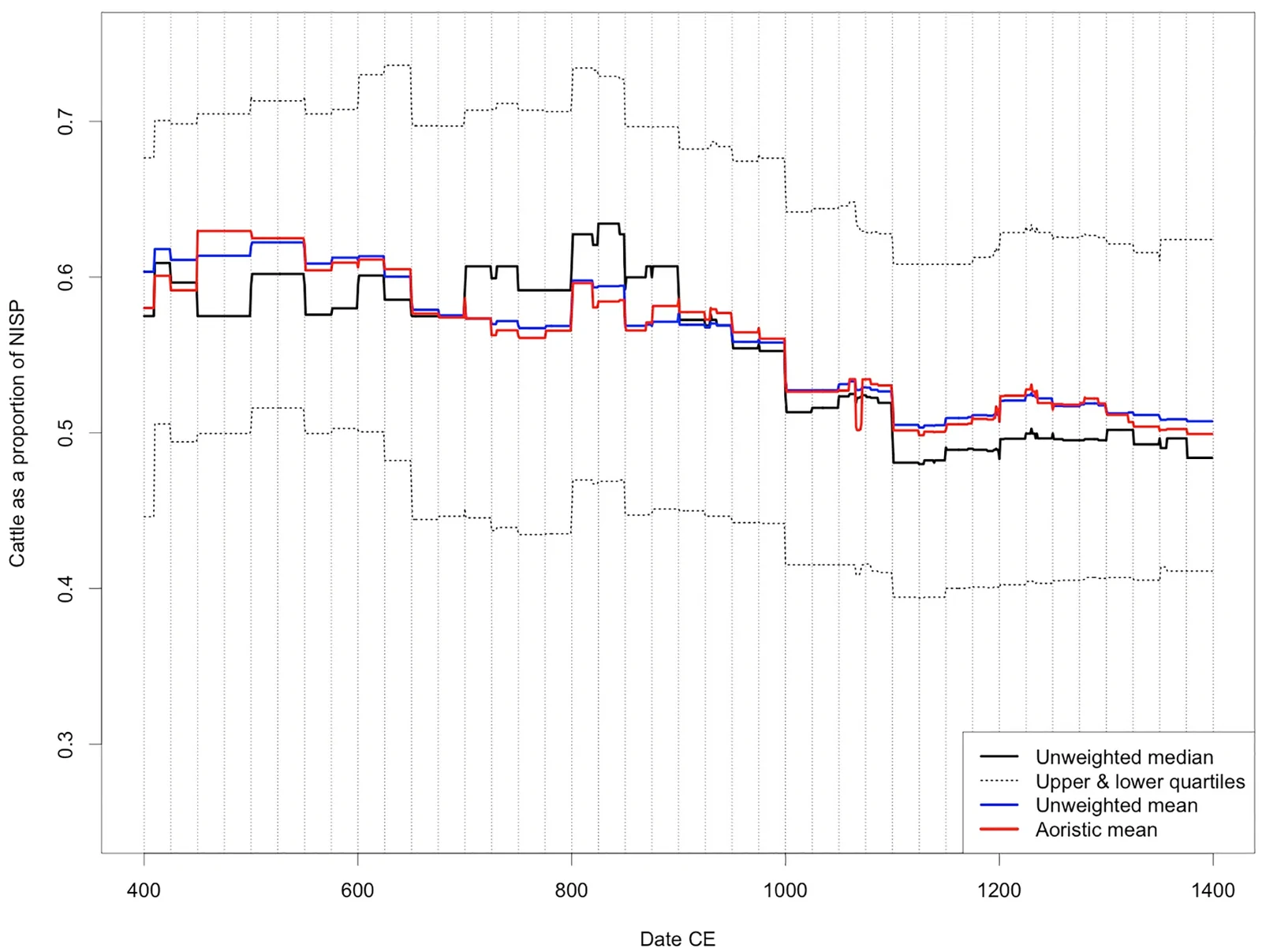
Comparison of different methods for calculating average contribution of cattle through time, as a proportion of total cattle and sheep NISP.

Given the overall similarity in results (Fig 3), we favour the unweighted approach for present purposes on the grounds of greater simplicity and transparency, combined with the ability to visualise dispersion (in this case interquartile range) at each snapshot in addition to central tendency. In other situations, for example where there is greater variation in phase duration, one might expect greater divergence between the methods.

### Site types

The mobile nature of animals in the landscape means that they can be easily moved between sites. High-status and ecclesiastical settlements may be expected to draw on animals from farms within their estates, while urban consumers would have obtained livestock from the surrounding hinterland and potentially further away. Even rural sites would not have been exempt from the redistribution of animals, which were probably subject to movement for several reasons, such as breeding, transhumance, trade and exchange. It should be noted that the movement of preserved or transformed meat products such as sausages or salt beef would not include bones, and these products will be invisible in the archaeological record at the site of consumption. Although the bulk of analysis carried out for this investigation considers data from all sites, they were also classified as ‘urban’, ‘rural’, ‘high-status’ and ‘ecclesiastical’ to understand the demands of specific site types on the production, redistribution or consumption of cattle and sheep (Table 2).

The concept of an urban settlement is complex and intangible [67] and in this study they are distinguished as settlements for which the majority of inhabitants were not engaged in purely agrarian activities; mid-Saxon *wics* and late Saxon *burhs* are both included in this category. Sites located in former Roman towns in the immediate post-Roman period are also included as being distinct from rural populations.

### Mortality data

The eruption and wear of teeth was used to discern the age at slaughter, since it provides an indication of age at death for the entire lifespan, in contrast to epiphyseal fusion that stops when the animal has finished growing. Tooth wear data were recorded when the specialist incorporated systems that produced wear stages (following Grant [68], Halstead [69], or Payne [70]), or provided raw data that could be converted into wear stages. Table 3 provides details on what ages the wear stages are likely to correspond to. However, methods of absolute ageing are flawed, observed in the differences between the studies presented in Table 3 (for a good summary see [73]), so the results of this analysis will be largely described using the broad descriptions of O’Connor [73], or the wear stages themselves.

**Table 3:** Mandible wear stages for cattle and sheep and their approximate corresponding ages (in months) commonly cited, the exception is Grant, who provides the equivalent Mandible Wear Stage (MWS). Square brackets indicate author’s terminology.

Wear stage	Sheep				Sheep and cattle	Cattle		
	Jones [71]	Payne [70]	Greenfield & Arnold [72]	Grant MWS [68]	O’Connor [73]	Halstead [69]	Jones & Sadler [74]	Grant MWS [68]
A	<1	0-2	0-2	1-2	Neonatal	0-1	0	1-3
B	1-3	2-6	2-6	3-7	Juvenile	1-8	0-6	4-6
C	3-12	6-12	6-12	8-18	Immature	8-18	5-18	7-16
D	10-24	12-24	12-24	19-28	Sub adult	18-30	16-28	17-30
E	23-36	24-36	24-36	29-33	[young] Adult	30-36	26-36	31-36
F	36-66	36-48	36-48	34-37	Adult	Young adult	34-43	37-40
G	66-96	48-72	48-72	38-41	[old] Adult	Adult	40-78	41-43
H	96-120	72-96	72-96	42-44	Elderly	Old adult	60-120	44-45
I/J	>120	96-120	96-120	>45	Elderly	Senile	72-192	>46
K	–	–	–	–	–	–	168-240	–

An aoristic approach was employed for mortality data, including data from all sites with ten or more tooth wear stages recorded, at 25-year intervals. The mean proportion of wear stages were calculated for each assemblage, so not to bias the results towards sites with large numbers of mandibles. The mean proportion of animals present at each wear stage from all assemblages is illustrated.

In a subsistence economy in which animals die at a range of ages, some as birthing casualties, some culled for meat, some later for purposes of small-scale milking, wool supply or traction, the proportion of animals at each stage will be evenly spaced, as there is no emphasis on a particular product. When the economy requires a greater demand on a specific product, for example if there is an emphasis on young animals culled for meat or older animals used for secondary products, then one or more age stage will be disproportionately represented.

One problem in the interpretation of mortality data is the effect that longevity in animals will have on quantification [11,75]. For example, a prized milking cow or draught oxen kept alive until they are nine years of age will present as one carcass in the zooarchaeological record, while potentially three beef animals could have been raised and culled in the same period, inflating the number of subadult or young adult animals in comparison to one working animal. It should therefore be remembered that in a mixed economy the number of older animals observed in the death record will be lower compared to those raised for meat at any one time while alive.

### Sexing data

Information regarding the numbers of male and female animals was patchy. The methods used to inform the sex of cattle and sheep in site reports were highly varied, often amalgamated into the discussion of the site rather than quantified by phase. Characteristics used to sex populations included morphological observations of various elements, most commonly pelves, metacarpals and horn cores. Sex data from cattle and sheep pelves and cattle metacarpals were also recorded during re-analysis of several assemblages as part of the *FeedSax* project. Sexual characteristics of sheep and cattle pelves are defined by the height of the acetabular rim and morphology of the pubis [76–78], and those of cattle metacarpals by size and shape [79]. The inclusion of two elements for cattle has implications for discerning the underlying animal husbandry, as their pelves fuse between 7 and 10 months, earlier than metacarpals that fuse between 24 and 30 months [80]. Animals that are culled early in life will therefore be observed in the pelvis but not the metacarpal data, which can aid understanding of sex-specific culls.

Castrated males are harder to observe and are rarely identified archaeologically as their metapodials are often less robust but longer than bulls or rams and measurements of castrated male sheep pelves plot between and overlapping those of females and males [78]. It is likely that most adult males identified will be castrates, as these are easier to handle and can be kept in mixed herds or flocks with cows and ewes all year round.

Translation of these trends into absolute end products is also ambiguous, made more complex by the problem of equifinality [81]. For example, while an emphasis on older male sheep is likely to reflect the importance of wool production, given that they cannot also be used for dairy and are not required in large numbers for breeding purposes, it is harder to argue the case for specialist production from a predominantly female flock that has potential to produce milk, wool and young. The same is true for cattle: oxen are likely to be used predominantly for draught purposes, but cows can be used for a combination of traction, milk and breeding.

### Geology

The superficial geology of each site was recorded and placed into one of five categories: chalk (including chalk alone, or with flint, sand and/ or gravel), clay (including clay with sand, loam, alluvium, brickearth, flint, boulder clay, silt and/ or gravel), valley terrace (any mixture of alluvium, silt, sand and gravel) and sand. Settlements on mixed geologies (i.e., chalk and clay and/ or alluvium) were excluded from comparisons, as were urban sites as it is likely that animals were brought to such sites from non-local farms.

## Results

**Fig 4:**
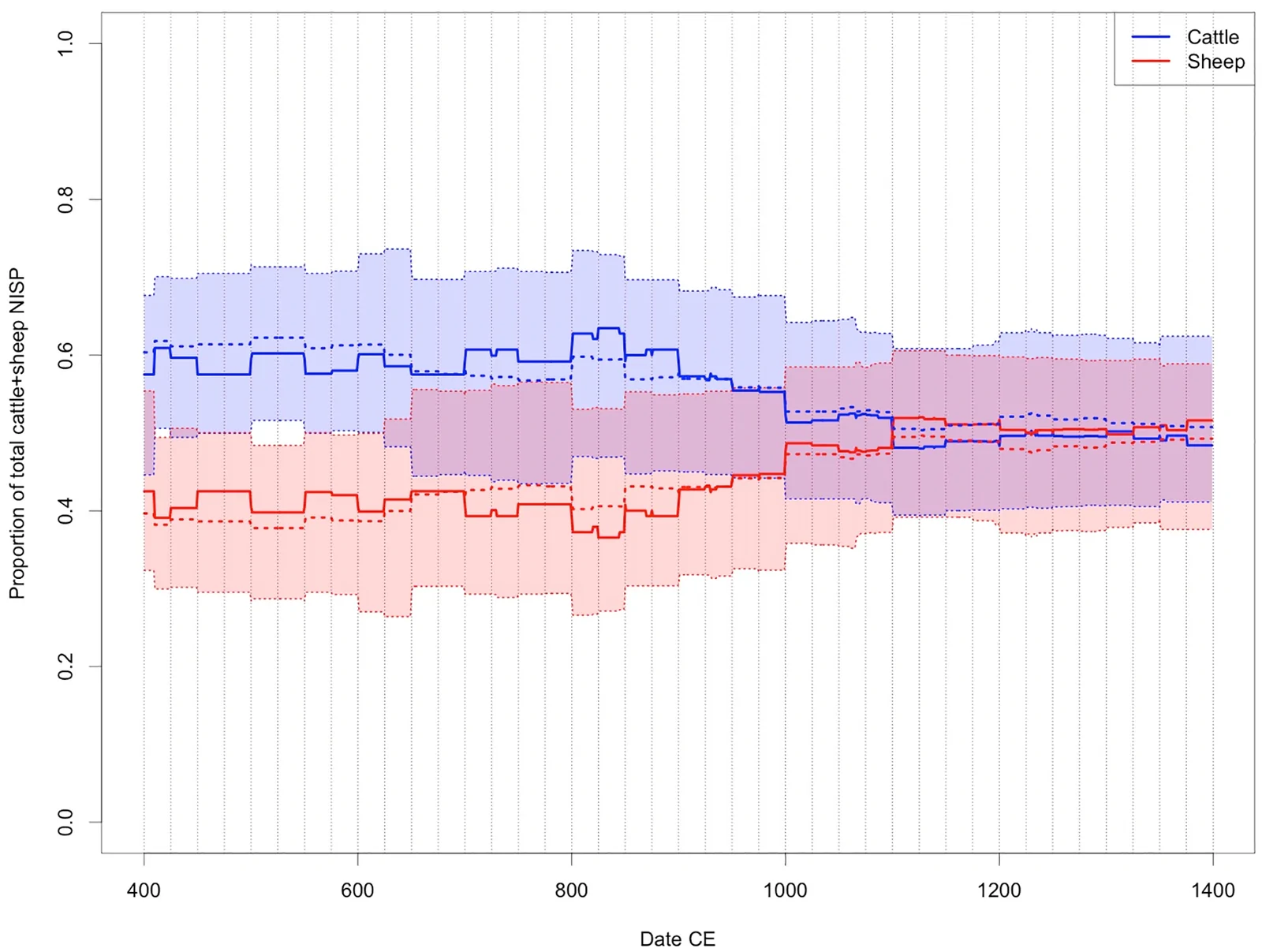
Relative contributions of cattle and sheep NISP through time: Unweighted mean (dashed line) and median (solid line). Shaded areas represent the inter-quartile range.

**Fig 5:**
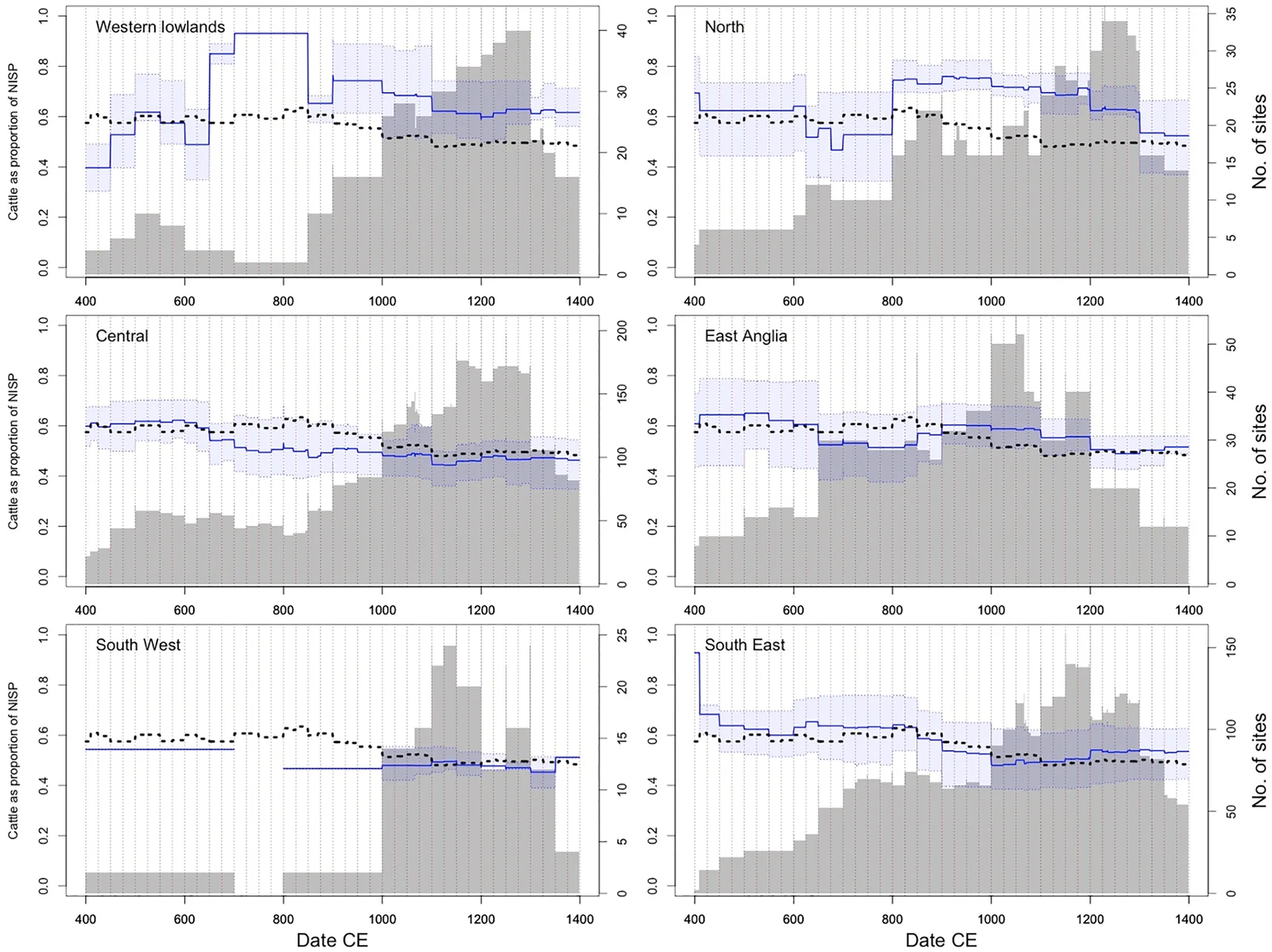
Trends in contribution of cattle to total cattle and sheep NISP across all sites, by region [63]. Solid blue lines represent unweighted mean; blue shaded areas represent inter-quartile range; dotted black line shows overall trend across all regions for comparison. Grey shaded bars show number of sites contributing at each point in time. Periods with fewer than 5 sites should be treated with caution during analysis.

**Fig 6:**
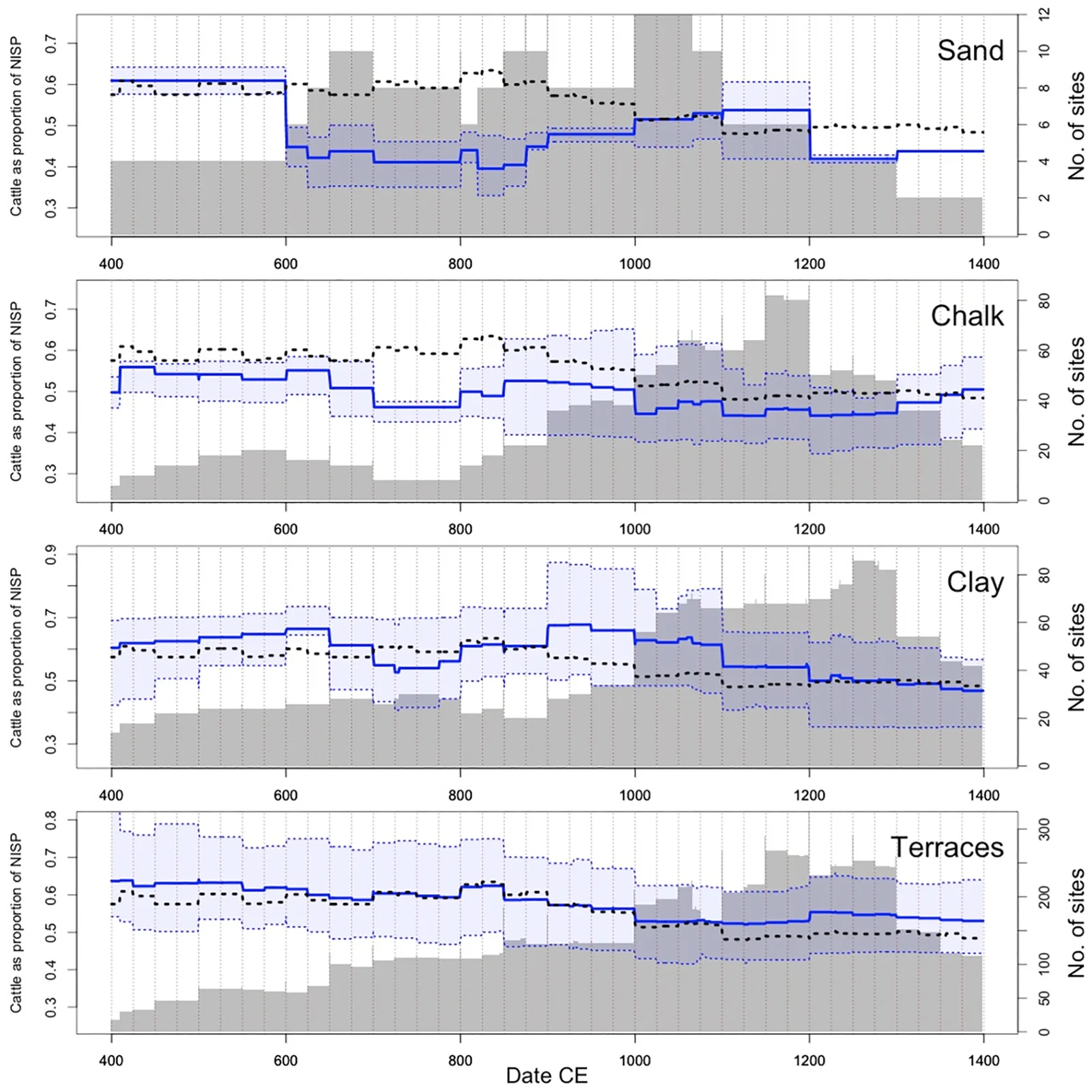
Trends in contribution of cattle to total cattle and sheep NISP across all sites, by geology. Solid blue lines represent unweighted mean; blue shaded areas represent inter-quartile range; dotted black line shows overall trend across all geology types for comparison. Grey shaded bars show number of sites contributing at each point in time. Periods with fewer than 5 sites should be treated with caution during analysis.

**Fig 7:**
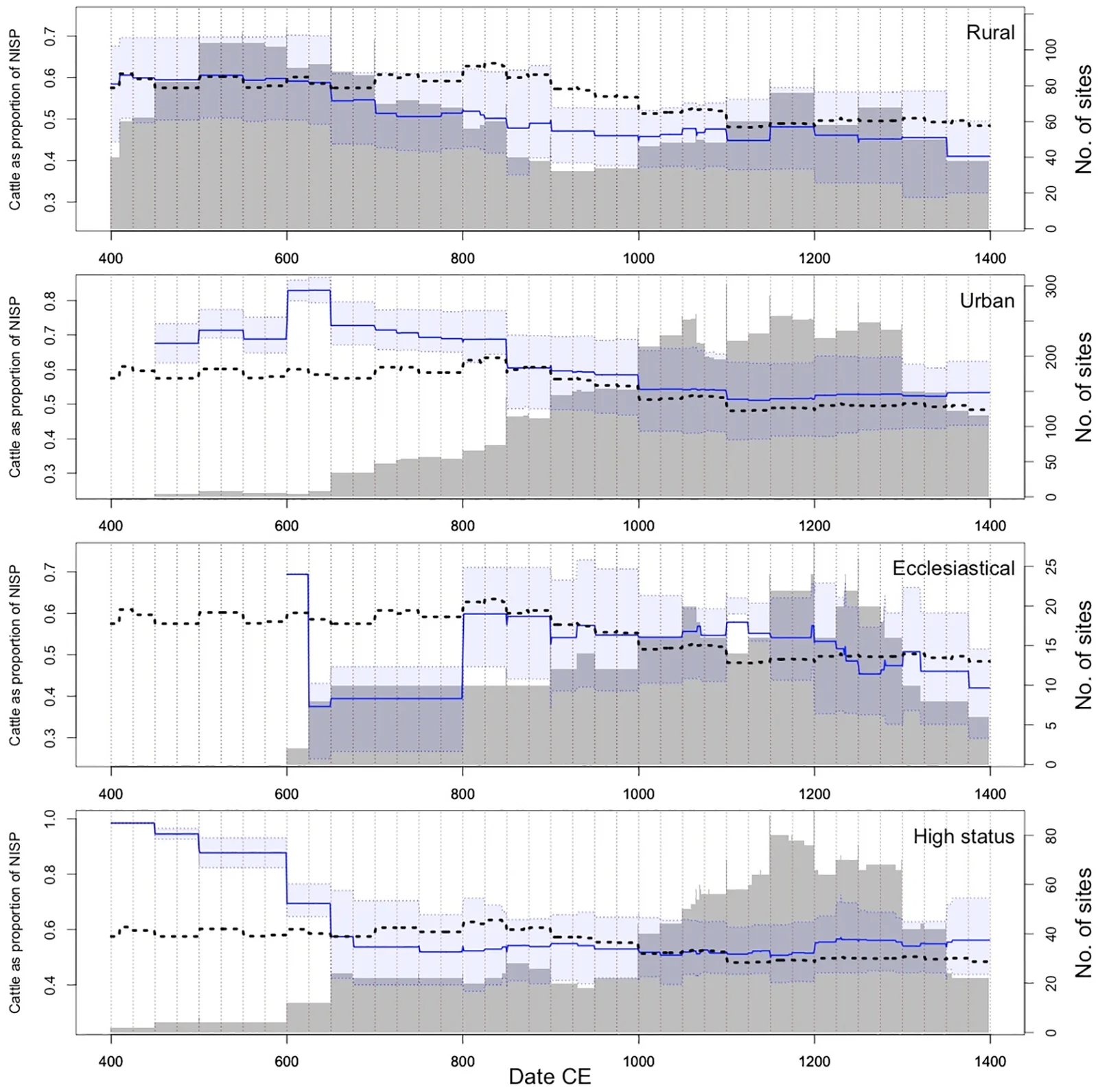
Relative proportion of cattle and sheep by site type (‘urban’ sites include Roman towns, *wics* and burhs). Solid blue lines represent unweighted mean; blue shaded areas represent inter-quartile range; dotted black line shows overall trend across all site types for comparison. Grey shaded bars show number of sites contributing at each point in time. Periods with fewer than 5 sites should be treated with caution during analysis.

Fig 4 shows the unweighted continuous temporal mean for cattle and sheep proportions in the overall dataset. Results for species proportions by region, site type and geology are presented in a series of charts (Figs 5-7), based on the relative proportions of cattle and sheep compared to the overall mean described in Fig 4. Trends are considered notable if they fall outside the quartile ranges (the blue bands). The number of sites from each region is also provided so that small sample sizes (e.g., fewer than five sites) can be treated with caution.

### Cattle and sheep

Cattle are the most common of the two main domesticates at all sites during the period CE 450–625 (Fig 4). The proportion of sheep begins to creep up from CE 625, albeit with another increase in cattle between CE 800 and 850. From CE 850 the proportion of sheep increases until CE 1000 when the two taxa are commonly recorded in parity, a trend lasting until the end of the study period at CE 1400. Despite the similarity in numbers, it must be remembered that cattle will produce far greater yields of meat, milk and raw materials than sheep and therefore the former contribute more to the economy.

There is no difference observed between regions with large enough sample sizes until CE 700, when the proportion of sheep recorded in the central region is at the upper limits of the overall inter-quartile range, lasting until CE 1000 (Fig 5). From CE 800 cattle are dominant in the north and, from CE 900 the west of the country, a trend that continues until CE 1300; and from CE 1000 until CE 1200 cattle are more common in East Anglia, though to a lesser extent than in the north and west. There is also some evidence for environmental influence, whereby sheep are more common on lighter sandy and chalk soils, until CE 1100 and 850, respectively (Fig 6).

A word of caution is necessary here: the high proportion of cattle recorded in the north of the country from the ninth century has previously been interpreted as a cultural preference of the Scandinavian diaspora [82,83]. Yet this trend potentially begins earlier than the major incursions of the mid-ninth century and most likely reflects a combination of an absence of assemblages on chalklands and rural sites (only two such settlements being recorded between the ninth and fourteenth centuries) that are traditionally the home of sheep, and poor preservation [66] that may lead to an under representation of smaller bones. A similar argument can be made for the high proportion of cattle observed in East Anglia and the western lowlands, as only one and two rural sites are represented, respectively.

Site type also appears to be a factor in the dominance of each taxon after CE 600, prior to which nearly all excavated sites are rural. Cattle are most common on high-status sites until CE 650, urban sites until CE 850 and ecclesiastical sites between CE 1100 and 1150 (Fig 7), while sheep are more likely to be found at ecclesiastical sites between CE 625 and 800 and, to a lesser extent, rural settlements between CE 700 and 1100.

### Cattle demography

**Fig 8:**
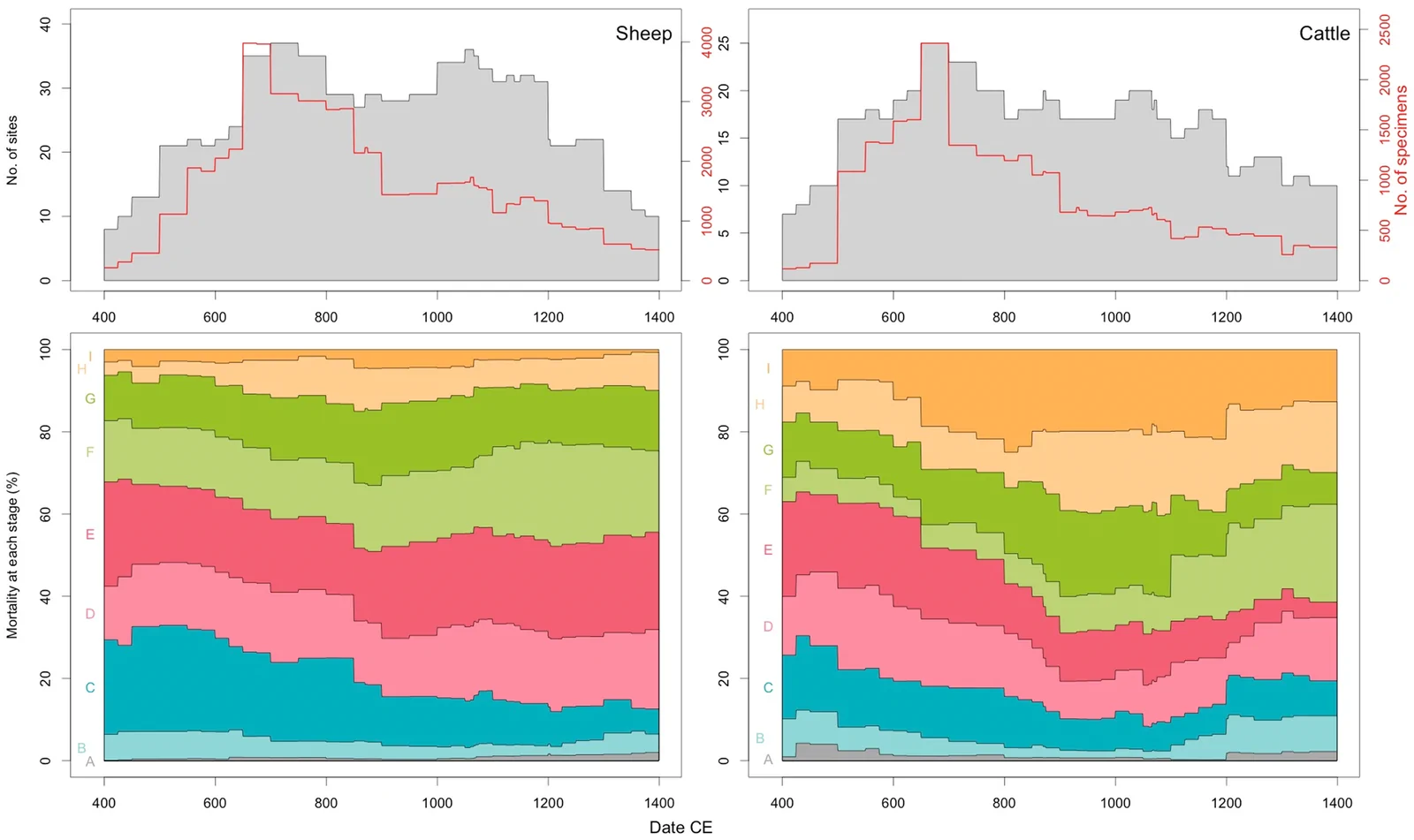
Cattle and sheep age data showing proportion of animals at each wear stage at 25-year intervals. All sites included where N > 9 mandibles. For approximate ages see Table 3.

The proportion of cattle culled at each wear stage is relatively evenly spaced between CE 400 and 650 (Fig 8), suggesting a self-sufficient economy, where animals were raised largely for meat, with some younger birthing casualties and some older animals used for small-scale milk and/ or draught work, a strategy that is not observed again during the medieval period.

From CE 650 an increasing proportion of cattle are culled later. This begins with elderly adults at stage I that move from representing c.10% to c.20% of the mortality data, followed by an increase in adults at stage G from CE 800, and older adults at stage H from CE 875, so that at the peak in this trend towards older animals between CE 900 and CE 1100, 60% of cattle are culled at wear stage G or later. This implies an increasing emphasis on secondary products (milk or draught work) in the economy. A further change can be observed from CE 1100, when more young adult cattle are recorded at wear stage F, increasing again from CE 1200, largely relative to a decrease in older adults at wear stage G. It should be noted that the proportion of younger animals at stages A to E and older adults at stages H and I remain similar between CE 900 and 1400, though the proportion of calves at stages A and B increase from CE 1200.

**Fig 9:**
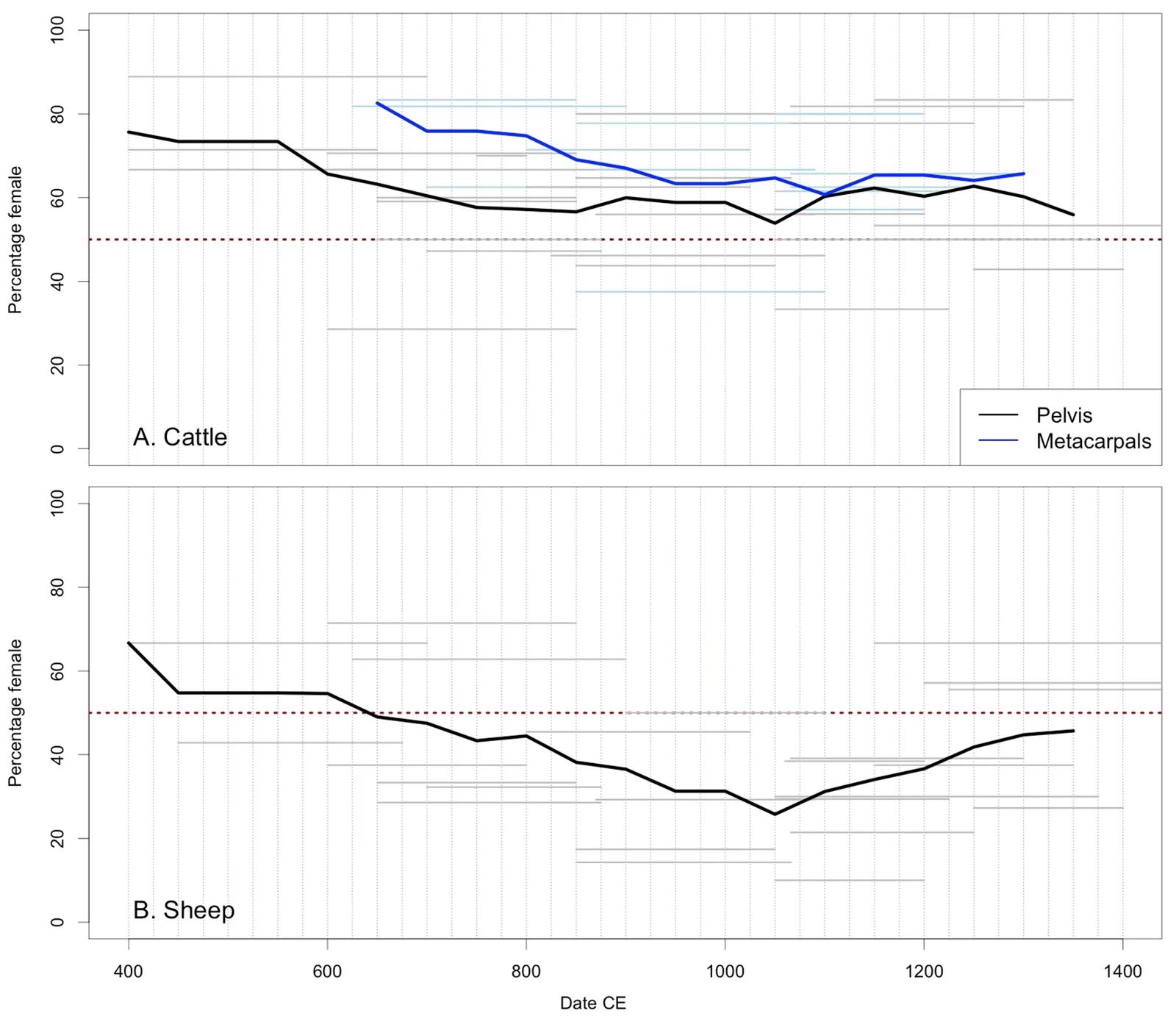
Proportions of female livestock remains, for cattle (A) and sheep (B). Horizontal bars represent observed female proportions in individual assemblages based on pelvis morphology (black) and metacarpal measurements (blue, for cattle only). Trend lines show unweighted means calculated at 50-year intervals (same colour coding). No attempt has been made to differentiate castrates from intact males.

Female cattle are predominant in the sex data in all periods, though males are more common in the pelvis data than the metacarpal data until CE 900 (Fig 9). As the pelvis fuses earlier than the metacarpals, this implies that males were preferentially culled before reaching c.30 months (when the distal metacarpal becomes fully fused).

**Fig 10:**
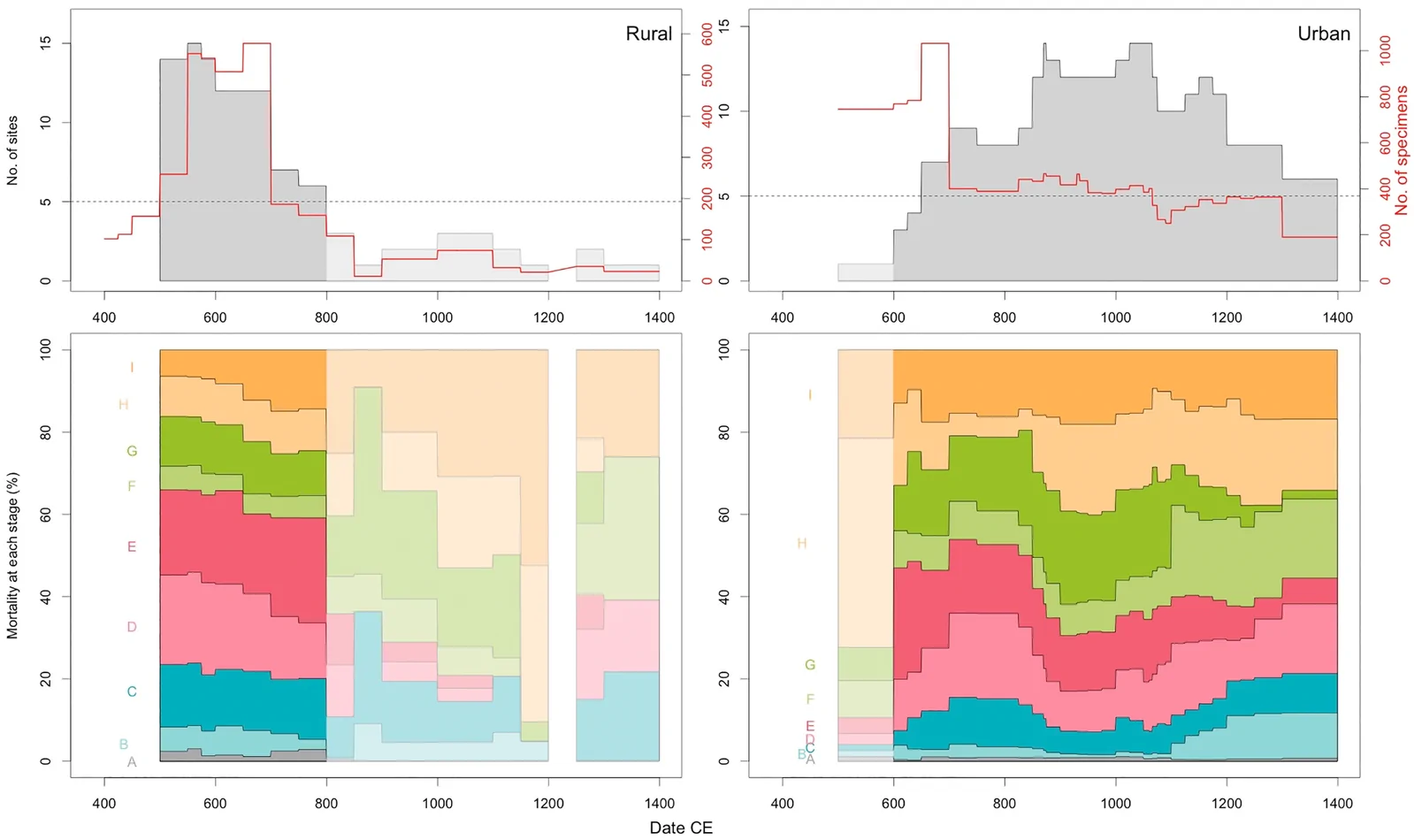
Cattle age data at rural and urban sites. Showing proportion of animals at each wear stage at 25-year intervals. Sites included where N > 9 mandibles, absent bands = no data, shaded areas <5 sites. For approximate ages see Table 3.

Investigations into the provisioning of urban sites with animals at various ages is hindered by the availability of suitable sample sizes for both rural and urban sites (Fig 10). It is only between CE 600 and 800 that the movement of animals between rural sites and *wics* can be explored. Cattle were slightly younger at rural sites, c.60% at wear stages A to E, corresponding to c.50% at wics. Slightly more very young animals at wear stages A and B were also recorded at rural sites.

A similar problem affected the investigation into the animal economy of different regions, and it was only possible to compare the central zone with other regions between CE 450 and 700, neither of which showed any difference in the ages of animals culled.

### Sheep demography

In all periods the sheep economy was stable, with animals culled at all ages (Fig 8). Some small adjustments can be observed, with an increasing emphasis on those used for secondary products at wear stages F and G occurring from CE 650. This peaked at CE 850 and continued until CE 1400, whereby c. 45% of all animals were culled at wear stage F or above. One further trend of note is the proportion of sheep that died young at wear stage C, which decreases from CE 850.

Sample sizes are extremely small for the sex data, but from CE 750 males are more commonly recorded, peaking at CE 1050 (Fig 9).

**Fig 11:**
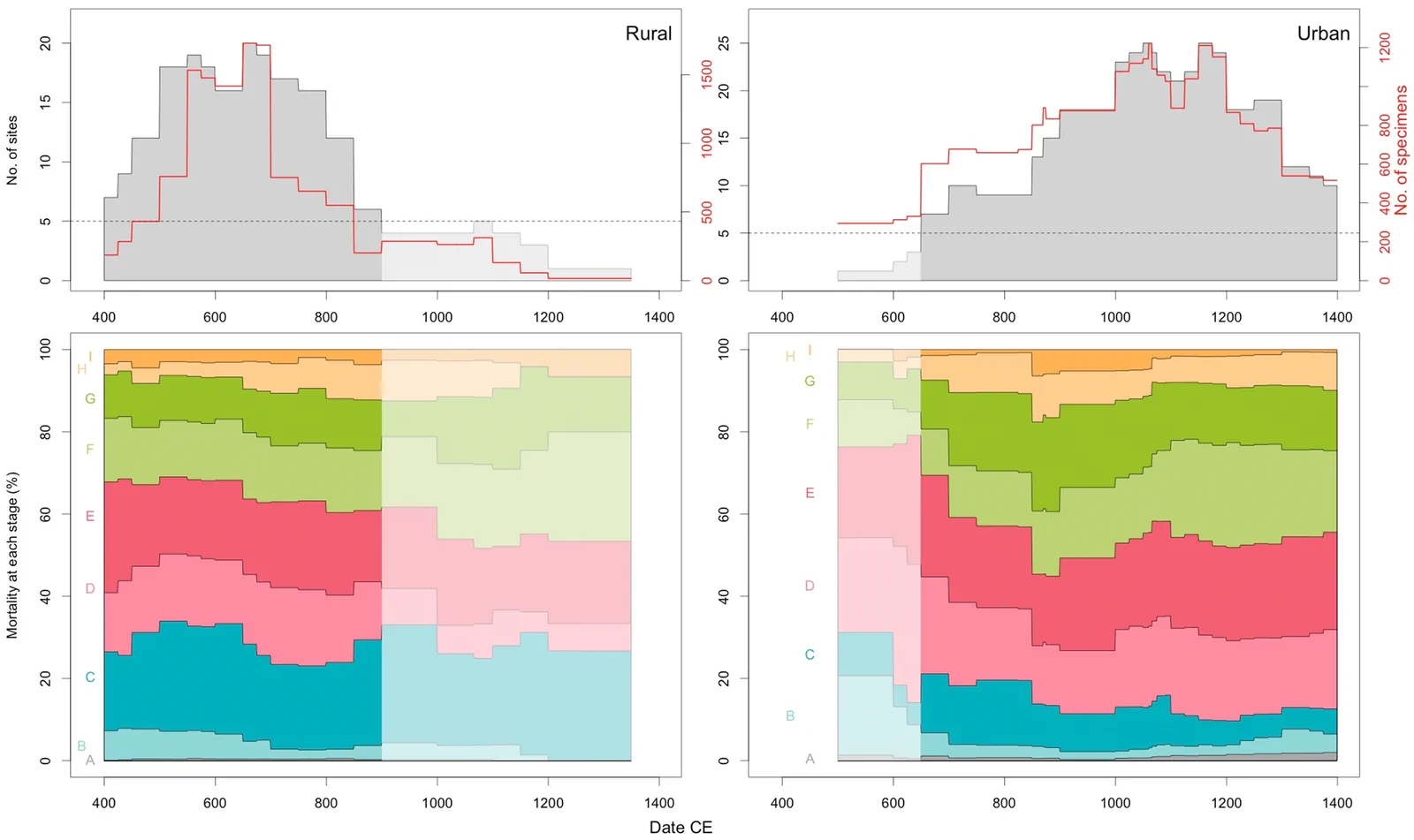
Sheep age data at rural and urban sites. Showing proportion of animals at each wear stage at 25-year intervals. Sites included where N > 9 mandibles, shaded areas <5 sites. For approximate ages see Table 3.

As with cattle, sample sizes affected the reliability of comparisons of sheep mortality data between urban and rural sites. The only suitable period was between CE 650 and 900 (Fig 11). Results showed little difference in the age of sheep recorded at rural sites over time, and the same is true of urban sites until CE 850, when c.55% of sheep at burhs were adults a wear stage F or above, compared to c.40% of those on rural sites.

**Fig 12:**
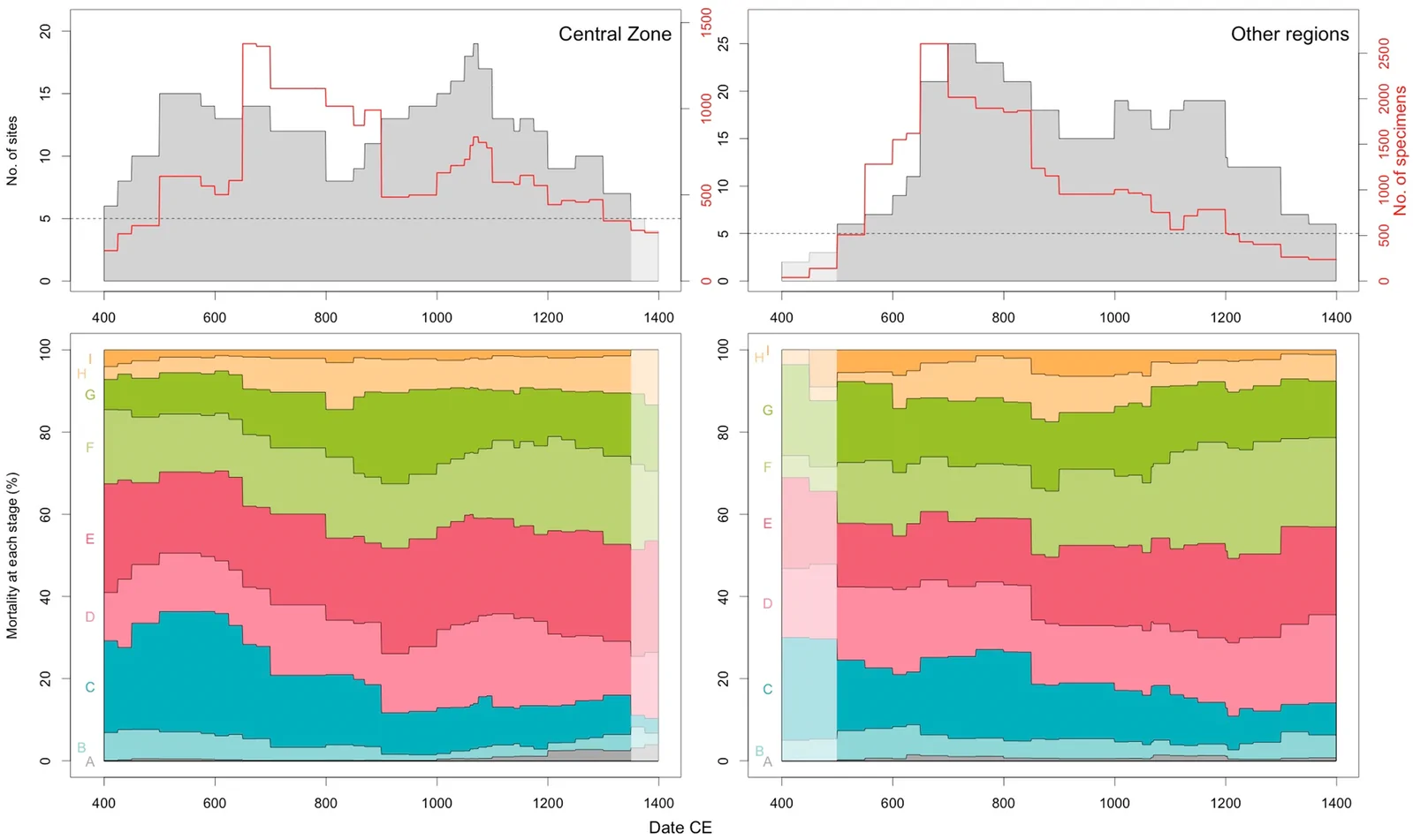
Sheep age data from the central zone and other regions. Showing proportion of animals at each wear stage at 25-year intervals. Sites included where N > 9 mandibles, shaded areas <5 sites. For approximate ages see Table 3.

When split between the central zone and other regions, sheep mortality data are recorded in large enough sample sizes for most of the study period (Fig 12). Results from outside the central zone vary little over time, though a slight increase in the proportion of a adults at stage F and above can be observed from CE 850, which is consistent with the overall mortality profile (Fig 8). Between CE 500 and 650, however, the central zone produced a greater proportion of younger sheep at wear stages C and E. At CE 650 the proportion of these younger animals decreases and the rest of the period is similar to the overall trend.

## Discussion

The results of this new analysis are considered alongside traditional narratives of the medieval animal economy, focusing on the broad hypotheses described previously. A summary of findings is presented in Table 4.

**Table 4:** Summary of the major periods of change observed in the animal economy.

Date range (CE)	Relative proportions	Cattle Husbandry	Sheep Husbandry	Summary
400-425	Mostly cattle	Mixed	Mixed, dairy	Post Roman continuity at some sites. Dairy cattle and sheep
425-450				
450-475				
475-500				
500-525			Mixed	Self-sufficient production. Small surplus
525-550				
550-575				
575-600				
600-625			Mixed, wool	
625-650	Mostly cattle; increase in sheep from 625; brief increase in cattle 800–850			More sheep, particularly in CZ starts with monastic influence. More older and male cattle, few draught cattle signatures
650-675		Increase in secondary		
675-700				
700-725				
725-750				
750-775				
775-800				
800-825				Wool and possible grain production high. Male cattle culled later. Peak older cattle 900–1100; peak draught cattle 1000–1200
825-850				
850-875			Wool	
875-900				
900-925		Focus on secondary, older males		
925-950				
950-975				
975-1000				
1000-1025	Cattle = sheep			
1025-1050				
1050-1075				
1075-1100				
1100-1125				
1125-1150				
1150-1175				
1175-1200				
1200-1225		Increase in meat		Meat production increasingly important. Fewer draught cattle, increase in dairy
1225-1250				
1250-1275				
1275-1300				
1300-1325				
1325-1350				
1350-1375				
1375-1400				

### H1 and 2: Mixed farming with a focus on cattle CE 450–650

The widely perceived reliance on a cattle-based, mixed economy in the post-Roman period has been confirmed, and the data illustrate the widespread and ingrained trend that represents the value of cattle as a form of portable wealth [84].

At this time most settlements revert to a self-sufficient mode of production centred on meat production with some small-scale wool, milk and draught use, but there is continuity of late Roman patterns at some sites. In most areas the late Roman rural animal economy is typified by high proportions of cattle, many of which were kept as adult or elderly animals [85,86]. This strategy can be observed between the fifth to seventh centuries at Fossets Farm, Southend and Market Lavington, Wiltshire, where over half the mortality data came from adult or elderly animals at wear stages G or H. Two assemblages (Market Lavington again, and Quarrington, Lincolnshire) also produced cattle remains identified with high ‘draught cattle’ signatures [25]. Draught cattle signatures are calculated using pathological and sub-pathological changes to cattle lower limb bones, particularly those of the hind limb (for more detail see [87,88]. In combination, this implies a continuation of arable production at some sites on a scale more typical of the late Roman period than post-Roman self-sufficiency. In his review of the animal remains from Central England, Albarella [7] also raises this possibility. This is increasingly observed in other aspects of archaeology, where research has implied continued high-status occupation of Chedworth villa well into the fifth century [89], field boundaries respected into the medieval period [11,90–92] and populations living within Roman towns such as Wroxeter and York [93–95]. However, it is important to note that evidence for continuity of Roman ways is not common, and most settlements see a down-scaling of agricultural production observed as a more self-sufficient regime with little evidence for movement of animals into or away from rural sites, reflecting the reduction in demand for surplus production. This resulted in greater variation in production, dependant on local conditions [91], reflected in the predominance of sheep on chalk downlands (Fig 6) where they would have been integral to arable farming on thin soils for providing nutrient-rich manure vital for crops [87,96]. Similar findings have been observed in pollen data for some regions, where a modest decline in land use and some increase in pasture and woodland occurs from the late Roman period [25].

Given the likely value of cattle within society at this time [84], it is perhaps surprising that so many were culled young, primarily for meat. The predominance of cows suggests they were preferentially selected as being easier to handle, and of greater value on a small farm where they could be used for milk, breeding and draught work. From the earliest data available it appears that young male cattle were deliberately culled over heifers, which further implies the availability of milk and dairy products in the economy.

The first move away from this picture comes in around CE 625, when an increase in sheep can be linked to the new monastic complexes, for reasons ranging from their allegorical use in scripture to their economic value [97]. Three of the top five sites with more than 65% sheep are monastic (Hartlepool Monastery, Church Close, Hartlepool and Eynsham) and the other sites are rural. The increase in sheep in the earlier 7^th^ century data from East Anglian, northern and central sites (Fig 5) further implies a widespread increase in sheep husbandry. This supports the suggestion that rural production was a driver of international trade and of the founding of *wics* in the mid-7^th^ century [98], rather than a response to a market demand for wool and cloth.

### H3: Increased local specialisation CE 650–850

From the mid-seventh century the animal economy became more complex as the increase in trade, both on a domestic and international scale, required surplus production of food and raw materials [99,100]. While the overall trend continues to reflect cattle present in the greatest quantities, this is particularly notable at *wics*. An increase in the proportion of sheep at rural sites and their continuing popularity at monastic sites reflects a provisioning network requiring the translocation of cattle away from rural and possibly monastic sites to these new trading settlements [9,21,101].

Changes in husbandry strategies are further evidenced by an increase in the proportion of older sheep in general, and males in particular, consistent with an emphasis on wool [5]. The importance of wool as an exported commodity can be observed in other aspects of archaeology, with loom weights and spindle whorls vital to the production of cloth being common finds on many contemporary rural sites [102]. The scale of cloth manufacture increases from one of household production to the use of workshops associated with estate centres and wics [103]. Documentary evidence for the period further implies that English cloth exports were in the category of high quality, bulk goods, of sufficient significance to be mentioned in a letter dated CE 796 from Charlemagne to Offa of Mercia [104], document 197.3.1, albeit in a complaint about falling standards. Wool had a value of two pence per fleece at this time, compared to sixpence for a cloak or blanket and a shilling for a sheep and her lamb as described in the Laws of Ine in the late 7th century [104], document 32.2.2. While growing fleeces, ewes would also have produced milk, and it is likely that sheep’s milk was more important to cheese production than cow’s milk [105].

The increase in sheep in the central zone from CE 700 (Fig 5) cannot be discounted by the nature of the data (see cautionary note above) given the combination of urban and rural sites and large swathe of clay lands in this region. The contribution of sheep to the wool trade has already been noted, but the focus on sheep in the central zone has further implications for their role in arable systems as mobile manure units, weed control on fallow land, and the ability to thrive on and easily be moved to poorer soils away from crop-producing areas [5,105,106]. The archaeobotanical evidence from the *FeedSax* project further emphasises the importance of farmers in the Central Zone as early innovators in arable production [25].

Cattle assemblages continue to be dominated by females (Fig 7) and the draught use of cattle is neither intensive nor widespread in this period [25], which suggests that although grain production was increasing, it did not for the most part require cattle to be worked exceptionally hard or on heavy soils: most grain could be produced using an ard and/or on light soils. Exceptions exist, however, and high draught cattle signatures have been observed at Cook Street, Southampton, Sedgeford, Norfolk and Quarrington, Lincolnshire, all of which are on clay or valley terraces [25], which potentially represent animals that were part of a grain-producing economy that utilised heavier, more fertile soils. It is notable that these sites are all in the eastern and southern regions that were the focus of international trade through *wics* and potentially reflect the supply of surplus from the hinterland. This argument is strengthened when consideration is taken of the greater proportion of older animals recorded at *wics*, which implies the supply of such sites with larger, mature animals that would have produced the maximum amount of meat, and may have been less desirable than younger animals that had spent their lives in a field, that were possibly kept back deliberately by the famers of the rural hinterland.

### H4: Regional rise in wool production from c.CE 800–1200

Little change in the patterns of animal husbandry has previously been observed between the ninth and twelfth centuries [23] and while this is largely confirmed by this study, some exceptions have been identified.

The increase in sheep has been demonstrated earlier than the often-quoted ninth century, with its roots in the monastic estates of the seventh century. Sheep numbers continue to increase with time, and when coupled with the presence of older sheep and a greater proportion of wethers, reflect the continued importance of wool in the economy. The increase in older sheep in the ninth century provided a basis for the English medieval wool trade of the twelfth century [107–109] and explanations for the increase in imported silver coinage in the ninth century have been linked to the export of wool [1,110]. Textile manufacture moved into *burhs* in the ninth century, although it continued to be carried out on a household scale in rural settlements, production that increased in magnitude until it became an industry based on guilds in the twelfth century [103]. Though there is no indication for selective breeding of medieval sheep [109], the quality of wool varied by region, and by the fourteenth century the best came from the Cotswolds and Welsh Marches and the worst from the north of England and Scotland [111]. While half the nation’s flock was kept for wool, c.50% were less than two years old, culled for meat as they approach adulthood, reflecting a considerable surplus and the demand for lamb in the diet.

It is easy to point to sheep as producers of wool, but less obvious is their role within the agricultural economy as sources of manure, grazers of stubble and easily transported supplies of meat. This is exemplified by the peak and subsequent stability in the proportion of sheep in the eleventh century when grain production was critical for supporting an increasing population. The importance of sheep to the economy is reflected in the rights of the shepherd in the late 11^th^-century *Rectitudines Singularum Personarum* [112], where it states, “A shepherd’s due is that he should have 12 nights’ dung at Christmas, and 1 lamb from the year’s young ones, 1 bell-wether’s fleece, and the milk of his flock for a week after the equinox, and a bowl-full of whey or buttermilk all summer”, illustrating the value placed on all products (manure, wool and milk), in addition to meat.

There is little variation between site types from the tenth century (Fig 6), although sheep continue to be slightly more common on rural sites until CE 1100. The mid-9th century also brought changes in the provisioning of urban sites. Occupation of *wics* declined in response to Viking incursions, in favour of the well-defended *burhs*. Whereas the *wics* were most likely provisioned via the estate centres of the elite, *burhs* were supplied by meat and raw materials via a market, allowing more flexibility in the nature of the meat diet [113]. Consequently, cattle no longer dominated urban assemblages, as urban populations had more autonomy in what they could consume.

The predominance of sheep in the Central Zone continues until the eleventh century and, when combined with the high draught cattle signature in this region [25] and predominance of open fields [90,114], reinforces the view that this region was a focus of arable production.

The emphasis on grain production from the eleventh century implied by increasing sheep numbers is further reinforced by the presence of older, male cattle and a peak in ‘draught cattle’ signatures. Gradual changes in the rural economy that began in the seventh and eighth centuries converged in the tenth to twelfth centuries, driven by a period of population expansion, greater social hierarchy and urban markets that provide opportunities for agricultural output on a scale that had not been observed before in the medieval period, facilitated through a combination of crop rotation, mouldboard plough use, extensification and scaling up of land use [25].

### H5 and 6: Growing demand for meat and dairy from cattle from c.CE 1300 and a move away from grain production to meat, milk and wool from c.CE 1348

A shift towards early culls of cattle as they approach maturity implies an emphasis on beef from the twelfth century, earlier than previously recognised, and the increase in calves in the thirteenth and fourteenth centuries is consistent with an intensification of dairy production [115]. There is nothing to indicate regional, environmental or site-specific specialisation from the twelfth century as production and markets become more standardised. This represents a profound change, with a shift in emphasis away from older cattle towards a more diverse age profile, where young beef stock were kept in combination with fewer adults, although some do continue to be used for traction [25]. While the increasing urban populations would have required more beef for the table, the greatest impetus for the decline in working cattle is most likely to be the growing role of draught horses in the economy. The number of working horses increased from c.5% of all draught animals at the time of Domesday to c.20%−50% by CE 1400 [5,116].

Despite the increased culls of slightly younger sheep, the wool trade continued to prosper [111,117], the mid-fourteenth century French text, *Le Bon Berger* (the good shepherd) notes that “the reason and primary cause of the utility and profit [of sheep] is very clear… first of all clothes are made from the wool” [106] and this is reflected in the proportion of sheep recorded.

### Meat, milk, wool and grain: conclusions and further work

Analysis of the national dataset confirms many of the established trends in medieval animal husbandry, providing better understanding of the timing of several of these periods of change. Furthermore, the national database allows a consideration of trends for large parts of England, building a picture of the underlying animal economy, regardless of where cattle and sheep were taken to be culled (e.g., urban versus rural). Sex profiles are inconsistent and often unquantified in many reports and are hindered by low sample sizes at the site level. Future work should aim to standardise the methods used to sex the main domesticates and ensure full recording.

Much inter-site variation also exists, and although this has been explored by region and site-type, the data vary enormously between neighbouring sites, and even assemblages within the same settlement, as at Winchester [52] and Wallingford [118]. Geology and supply networks have also been shown to influence local decisions with an association between sheep, light soils and rural sites until the twelfth century representing the longstanding practice of farming sheep on the chalklands and the consumption of sheep at producer sites, while cattle were more often marketed to urban sites.

While existing hypotheses have largely been confirmed by the results of this analysis, new findings have been put forward that push at these boundaries and provide new research topics that should be further investigated in future:

1 **Meat**: Although livestock were inevitably part of the diet at the end of their lives, a demand for beef and lamb from younger animals culled at prime meat age can be observed in the twelfth century urban market. This occurs some two or three centuries earlier than previously described. While it is most likely due to the displacement of cattle by working horses, it is little understood and should be the focus of future research.
2 **Milk**: Dairy production has been the most difficult part of the animal economy to identify. The preferential keeping of female cattle and sheep in the early part of the study period implies that both cow’s milk and sheep’s milk were important and would have been readily available on most farms. By the thirteenth century an increase in calves at wear stage B implies the rise in dairy production, again somewhat earlier than the widely recognised fourteenth century veal trade.
3 **Wool**: The origins of the medieval wool trade have been firmly placed with the monastic communities of the seventh century, potentially acting as one of the drivers of international trade leading to the foundation of *wics*. Wool increases in importance again from the ninth century, in keeping with the accepted pattern of production.
  Despite an increase in the age of sheep from the ninth century, relatively few elderly sheep were observed that would have produced fleeces over many years; even at the peak of wool production, over half the animals were culled before reaching four years of age. Further investigation of the role of equifinality in intensive husbandry regimes may refine the interpretation of mortality data in future.
4 **Grain**: Though most sites in the immediate post-Roman period began to exhibit the type of self-sufficient farming that was to continue into the seventh century and beyond, a few managed to maintain late Roman patterns of arable production, indicating a long-lived strategy at a small number of settlements. A subsequent emphasis on arable production occurs from the seventh century in the Central Zone, spreading to all regions by the ninth century, observed in the increasing proportion of sheep and focus on cattle for draught work. The proportion of cattle living beyond their fourth year between the ninth and twelfth centuries was unexpected, and highlights the widespread importance of grain production at this time.
5 **Provisioning**: Sample sizes remain small for some mortality data, often making comparisons between site types and regions unreliable. Despite this, there is some indication for the provisioning of *wics* with older cattle, and burhs with older sheep, but greater resolution is needed, which may be possible if more detailed studies are undertaken centred around a single urban site and its hinterland.

From beginnings largely based on self-sufficient production in the post-Roman period, the medieval animal economy in England gradually combines greater production from cattle and sheep to bring about surplus creation on a scale that can support international trade, urban networks and an overall population of several millions. Small changes over long periods in the type of livestock kept gradually shifted the emphasis away from meat to one where wool, traction and milk are of greater value. Some larger-scale transformations occur, which bring about more critical change: In the seventh century, monastic sheep farming provides a surplus that potentially underpins the establishment of *wics* and international trade, and the ninth century sees a greater focus on wool and arable production. The utilisation of draught cattle peaks between the eleventh and thirteenth centuries, after which the use of horses for traction and demand for younger beef and cow’s milk from urban markets shifts the emphasis back to a focus on meat.

### Preface

This paper began as a supplementary addition to the ‘Feeding Anglo-Saxon England’ (*FeedSax*) project. It was intended as a platform for detailed analysis of the data that underpinned discussions of the pastoral economy and draught cattle in the project output [25,113,119,120], while allowing a wider consideration of the role of animals for meat, milk and wool within the animal economy. It became obvious early in the project that there were issues with the use of terms such as ‘early’ or ‘middle’ Saxon, and trying to fit data from many disciplines into pre-defined periods based largely on ceramic production. A paper by David Orton and colleagues on fish consumption [36] used a method that bypassed the need for periodisation, and this provided an intriguing prospect for analysis of synthetic data sets. A collaboration grew and additional data were compiled after the official end of the project, providing larger samples for the mortality data and later assemblages. The final offering has therefore become somewhat more than the sum of its parts, as methods have been refined and the data set expanded.

## References

[1] Dyer C. Making a living in the middle ages: The people of Britain 850-1520. London: Penguin. 2003.

[2] Hamerow H. Rural settlements and society in Anglo-Saxon England. Oxford: Oxford University Press. 2012.

[3] Griffiths D. The ending of Anglo-Saxon England: Identity, allegiance, and nationality. In: Hamerow H, Hinton D, Crawford S, editors. Anglo-Saxon archaeology. Oxford: Oxford University Press. 2011. p. 62–78.

[4] Broadberry S, Campbell B, Klein A, Overton M, van Leeuwen B. British economic growth 1270-1870. Cambridge University Press. 2015.

[5] Campbell BM. English seigniorial agriculture 1250-1450. Cambridge: Cambridge University Press. 2000.

[6] Hedges R. Anglo‐Saxon Migration and the Molecular Evidence. The Oxford Handbook of Anglo-Saxon Archaeology. Oxford University Press. 2012. p. 79–90. doi: 10.1093/oxfordhb/9780199212149.013.0006

[7] Albarella U. A review of animal bone evidence from central England. 61/2019. Portsmouth: Historic England. 2019.

[8] Coy J, Maltby JM. Archaeozoology in Wessex. In: Keeley H, editor. Env Archaeol: A regional review. London: Historic Buildings and Monuments Commission for England. 1987. p. 204–51.

[9] Holmes M. Southern England: A review of animal remains from Saxon, medieval and post medieval archaeological sites. 08/2017. Portsmouth: Historic England. 2018.

[10] Levitan B. Medieval animal husbandry in south west England: A selective review and suggested approach. In: Balaam N, Levitan B, Straker V, editors. Oxford: British Archaeological Reports British Series. 1987. p. 51–80.

[11] Rippon S, Wainwright A, Smart C. Farming regions in medieval England: The archaeobotanical and zooarchaeological evidence. Mediev Archaeol. 2014;58(1):195–255.

[12] Ryder M. Livestock remains from four medieval sites in yorkshire. Agric Hist Rev. 1961;9:105–10.

[13] Stallibrass S. Review of the vertebrate remains. In: Huntley JP, Stallibrass S, editors. Architectural and Archaeological Society of Durham and Northumberland Research Report. 1995. p. 84–198.

[14] Crabtree P. Middle Saxon animal husbandry in East Anglia. Bury St Edmunds: East Anglian Archaeology. 2012.

[15] Crabtree PJ. Sheep, Horses, Swine, and Kine: A Zooarchaeological Perspective on the Anglo-Saxon Settlement of England. Journal of Field Archaeology. 1989;16(2):205–13. doi: 10.1179/jfa.1989.16.2.205

[16] Maltby JM. Iron age, Romano-British and Anglo-Saxon animal husbandry: A review of the faunal evidence. In: Jones M, Dimbleby G, editors. Oxford: British Archaeological Reports British Series. 1981. p. 155–204.

[17] Crabtree PJ. Animal husbandry and farming in East Anglia from the 5th to the 10th centuries CE. Quat Int. 2014;346:102–8.

[18] Grant A. Medieval animal husbandry - the archaeozoological evidence. In: Clutton-Brock J, Grigson C, editors. Animals and archaeology 4 husbandry in Europe. Oxford: British Archaeological Reports International Series. 1984. p. 179–86.

[19] Astill G, Grant A. Animal resources. The countryside of medieval England. Oxford: Blackwell. 1988. p. 149–87.

[20] Holmes M. Animals in Saxon and Scandinavian England: Backbones of Economy and Society. Leiden: Sidestone. 2014.

[21] O’Connor T. Livestock and animal husbandry in early medieval England. QuatInt. 2014;346:109–18.

[22] Rizzetto M, Crabtree PJ, Albarella U. Livestock Changes at the Beginning and End of the Roman Period in Britain: Issues of Acculturation, Adaptation, and ‘Improvement’. Eur j archaeol. 2017;20(3):535–56. doi: 10.1017/eaa.2017.13

[23] Sykes N. The Norman Conquest: A zooarchaeological perspective. Oxford: British Archaeological Reports International Series. 2007.

[24] Sykes N. From cu and sceap to beffe and motton: The management, distribution and consumption of cattle and sheep in medieval England. In: Woolgar C, Serjeantson D, Waldron T, editors. Food in medieval England: Diet and nutrition. Oxford: Oxford University Press. 2006. p. 56–71.

[25] Hamerow H, McKerracher M, Bogaard A, Charles M, Forster E, Holmes M. Feeding medieval England: A long ‘agricultural revolution’, 700-1300. Oxford University Press. 2025.

[26] McKerracher M, Bogaard A, Bronk Ramsey C, Charles M, Forster E, Hamerow H. Digital archive for feeding Anglo-Saxon England (FeedSax): The bioarchaeology of an agricultural revolution 2017-2022. York: Archaeology Data Service. 2023.

[27] Hamerow H, Bogaard A, Charles M, Ramsey CB, Thomas R, Forster E. Feeding Anglo-Saxon England: The bioarchaeology of an agricultural revolution. Antiquity. 2019;93(368):1–4.

[28] Crabtree PJ. Agricultural innovation and socio-economic change in early medieval Europe: evidence from Britain and France. World Archaeology. 2010;42(1):122–36. doi: 10.1080/00438240903430373

[29] Crabtree PJ. Agricultural innovation and socio-economic change in early medieval Europe: evidence from Britain and France. World Archaeology. 2010;42(1):122–36. doi: 10.1080/00438240903430373

[30] Crabtree PJ. Early medieval Britain: The rebirth of towns in the post-Roman west. Cambridge University Press. 2018.

[31] Fisher A, Thomas R. Isotopic and zooarchaeological investigation of later medieval and post-medieval cattle husbandry at Dudley Castle, West Midlands. Environ Archaeol. 2012;17(2):151–67.

[32] Thomas R, Holmes M, Morris J. So bigge as bigge may be: tracking size and shape change in domestic livestock in London (AD 1220-1900). J Archaeol Sci. 2013;40(8):3309–25.

[33] Crema ER. Modelling Temporal Uncertainty in Archaeological Analysis. J Archaeol Method Theory. 2011;19(3):440–61. doi: 10.1007/s10816-011-9122-3

[34] Baxter MJ, Cool HE. Reinventing the wheel? Modelling temporal uncertainty with applications to brooch distributions in Roman Britain. J Archaeol Sci. 2016;66:120–7.

[35] Orton D, Morris J, Pipe A. Catch Per Unit Research Effort: Sampling Intensity, Chronological Uncertainty, and the Onset of Marine Fish Consumption in Historic London. Open Quaternary. 2017;3. doi: 10.5334/oq.29

[36] Orton DC, Morris J, Locker A, Barrett JH. Fish for the city: meta-analysis of archaeological cod remains and the growth of London’s northern trade. Antiquity. 2014;88(340):516–30. doi: 10.1017/s0003598x00101152

[37] Crema ER, Kobayashi K i. A multi-proxy inference of jōmon population dynamics using bayesian phase models, residential data, and summed probability distribution of 14C dates. J Archaeol Sci. 2020;117:105136.

[38] Albarella U, Pirnie T. A review of animal bone evidence from central England evidence. York: Archaeology Data Service. 2008.

[39] Holmes M. A review of animal bone evidence from the Saxon to post medieval periods in southern Britain. York: Archaeology Data Service. 2017.

[40] Holmes M. Food, status and complexity in Saxon and Scandinavian England: An archaeozoological approach. The University of Leicester. 2011.

[41] Noddle B. The animal bones. In: Shoesmith R, editor. Hereford City excavations volume 3: The finds. London: CBA Research Report. 1985. p. 84–95.

[42] Levitan B, Serjeantson D. The animal bone. In: Halpin C, Barclay A, editors. Excavations at Barrow Hills, Radley, Oxfordshire. Oxford: Oxford Archaeological Unit. 1999. p. 236–41.

[43] Baxter IL, Fane C. Animal and bird bones. In: Spoerry P, Atkins R, editors. A late Saxon village and medieval manor: Excavations at Botolph Bridge, Orton Longueville, Peterborough. Bar Hill: East Anglian Archaeology. 2015. p. 108–16.

[44] Hamilton-Dyer S. Animal bone. In: Preston S, editor. Reading and Windsor, old and new. Reading: Thames Valley Archaeological Services Monograph. 2005. p. 36–9, 114–20, 43–6.

[45] Holmes M. Animal remains. In: Nichol M, Massey R, Hart J, editors. Anglo-Saxon settlement on the Oxfordshire downs: Archaeological investigations at land north of the A417, East Challow. Oxoniensia. 2024. p. 404–13.

[46] Higbee L. Animal bone. In: Tuck A, editor. The archaeology of Hornsea project one offshore windfarm cable route: Agriculture, settlement, moats and saltworking in the Lincolnshire marshes. Salisbury: Wessex Archaeology. 2023. p. 255–68.

[47] Wilson B, Allison E. Animal bones and marine shells. In: Lambrick G, editor. Neolithic to Saxon social and environmental change at Mount Farm, Berinsfield, Dorchester-on-Thames. Oxford: Oxford Archaeology. 2010. p. Appendix 16.

[48] Homes M. The animal remains from Pathfinder House, Huntingdon. Archaeological Project Services. 2008.

[49] Holmes M. The animal remains. In: Hart J, Sworn S, Mudd A, Antrobus A, editors. Living and working in a medieval and later Bristol suburb: Excavations at Redcliff Quarter 2016-2018. Bicester: Oxford Cotswold Archaeology Monograph. 2025. p. 316–36.

[50] Hinman M, Spoerry P. The Still, Peterborough: Medieval Remains Between Cumbergate and Westgate. Cambridgeshire County Council Archaeological Field Unit. 1998.

[51] Thomas R, Vann S. Mammal and bird bones. In: Ratkai S, editor. Wigmore Castle, North Herefordshire: Excavations 1996 and 1998. Abingdon: Society for Medieval Archaeology Monograph. 2015. p. 145–71.

[52] Bourdillon J. Late Saxon animal bone from the northern and eastern suburbs and the city defences. In: Serjeantson D, Rees H, editors. Food, craft, and status in medieval Winchester. Winchester: Winchester Museums. 2009. p. 55–82.

[53] Albarella U. Pig husbandry and pork consumption in medieval England. In: Woolgar C, Serjeantson D, Waldron T, editors. Food in medieval England: Diet and nutrition. Oxford: Oxford University Press. 2006. p. 72–87.

[54] Albarella U, Dobney K, Ervynck A, Rowley-Conwy P. Pigs and humans: 10,000 years of interaction. Oxford: Oxford University Press. 2007.

[55] Hamilton J, Thomas R. Pannage, pulses and pigs: isotopic and zooarchaeological evidence for changing pig management practices in later medieval England. Medieval Archaeology. 2012;56:234–59.

[56] Maurer G, Evetts K, Matthews A, Chick J, Haxley L, Davies L. Multi-isotope evidence shows extensive free-roaming pig husbandry endured in medieval England. Front Environmental Archaeol. 2026;5:1–24.

[57] Orton D, Gaastra J, Linden MV. Between the Danube and the Deep Blue Sea: Zooarchaeological Meta-Analysis Reveals Variability in the Spread and Development of Neolithic Farming across the Western Balkans. Open Quaternary. 2016;2. doi: 10.5334/oq.28

[58] Albarella U. Tawyers, tanners, horn trade and the mystery of the missing goat. In: Murphy P, Wiltshire P, editors. The environmental archaeology of industry. Oxford: Oxbow. 2003. p. 71–86.

[59] Salvagno L. The neglected goat: A new method to assess the role of the goat in the English middle ages. Oxford: Archaeopress. 2020.

[60] Quantum GIS Development Team. Quantum gis geographic information system. Open source geospatial foundation project. http://qgis.osgeo.org/. 2019. Accessed 2014 January 14.

[61] Lowerre A, Lyons E, Roberts BK, Wrathmell S. Atlas of rural settlement in England. York: Archaeology Data Service. 2015. doi: 10.5284/1031493

[62] Roberts B, Wrathmell S. An atlas of rural settlement in England. London: English Heritage. 2000.

[63] Rippon S, Smart C, Pears B, Fleming F. The Fields of Britannia: Continuity and Discontinuity in thePaysand Regions of Roman Britain. Landscapes. 2013;14(1):33–53. doi: 10.1179/1466203513z.0000000005

[64] R Core Team. R: A language and environment for statistical computing. Vienna: R Foundation for Statistical Computing. 2023.

[65] Dowle M, Srinivasan A, Gorecki J. Data. Table: Extension of ‘data. Frame’. R package version. 2019;1(8).

[66] Baker P, Worley F. Animal bones and archaeology: guidelines for best practice. 2nd ed. Portsmouth: English Heritage. 2019.

[67] Perring D. Town and country in England: Frameworks for archaeological research. York: CBA Research Report. 2002.

[68] Grant A. The use of toothwear as a guide to the age of domestic ungulates. In: Wilson B, Grigson C, Payne S, editors. Oxford: British Archaeological Reports British Series. 1982. p. 91–108.

[69] Halstead P. In: Pryor F, editor. Ipswich: East Anglian Archaeology. 1985. p. 219–24.

[70] Payne S. Kill-off Patterns in Sheep and Goats: the Mandibles from Aşvan Kale. Anatol Stud. 1973;23:281–303. doi: 10.2307/3642547

[71] Jones GG. Tooth Eruption and Wear Observed in Live Sheep from Butser Hill, the Cotswold Farm Park and Five Farms in the Pentland Hills, UK. Recent Advances in Ageing and Sexing Animal Bones. Oxbow Books. 2015. p. 155–78. doi: 10.2307/j.ctvh1ds02.14

[72] Greenfield HJ, Arnold ER. Absolute age and tooth eruption and wear sequences in sheep and goat: determining age-at-death in zooarchaeology using a modern control sample. J Archaeol Sci. 2008;35(4):836–49.

[73] O’Connor T. The analysis of urban animal bone assemblages: A handbook for archaeologists. 19/2 ed. York: Council for British Archaeology. 2003.

[74] Jones GG, Sadler P. Age at death in cattle: Methods, older cattle and known-age reference material. Env Archaeol. 2012;17:11–28.

[75] Johannsen N. Draught cattle and the south Scandinavian economies of the 4th millennium BC. Env Archaeol. 2006;11(1):35–48.

[76] Davis S. The effect of castration and age on the development of the shetland sheep skeleton and a metric comparison between bones. J Archaeol Sci. 2000;27(5):373–90.

[77] Greenfield HJ. Sexing Fragmentary Ungulate Acetabulae. Recent Advances in Ageing and Sexing Animal Bones. Oxbow Books. 2015. p. 68–86. doi: 10.2307/j.ctvh1ds02.9

[78] Popkin P, Baker P, Worley F, Payne S, Hammon A. The sheep project (1): Determining skeletal growth, timing of epiphyseal fusion and morphometric variation in unimproved shetland sheep of known age, sex, castration status and nutrition. J Archaeol Sci. 2012;39:1775–92.

[79] Davis SJ, Svensson EM, Albarella U, Detry C, Gotherstrom A, Pires AE. Molecular and osteometric sexing of cattle metacarpals: A case study from 15th century AD Beja, Portugal. J Archaeol Sci. 2012;39:1445–54.

[80] Silver IA. The ageing of domestic animals. In: Brothwell DR, Higgs ES, editors. Science and archaeology. London: Thames and Hudson. 1969. p. 283–302.

[81] Marciniak A. The secondary products revolution, mortality profiles, and practice of zooarchaeology. In: Greenfield HJ, editor. Animal secondary products archaeological perspectives on domestic animal exploitation in the Neolithic and Bronze Age. Oxford: Oxbow. 2014. p. 186–205.

[82] Holmes M. We’ll have what they’re having, cultural identity through diet in the English Saxon period. Env Archaeol. 2016;21(1):59–78.

[83] O’Connor T. Livestock and deadstock in early medieval Europe from the North Sea to the Baltic. Environ Archaeol. 2010;15(1):1–15.

[84] Holmes M, Hamerow H, Thomas R. Close Companions? A Zooarchaeological Study of the Human-Cattle Relationship in Medieval England. Animals (Basel). 2021;11(4):1174. doi: 10.3390/ani11041174 33923977 PMC8073649

[85] Allen M, Lodwick L, Brindle T, Fulford M, Smith A. Agricultural strategies in Roman Britain. New visions of the countryside of Roman Britain volume 2: The rural economy of Roman Britain. London: Britannia Monograph Series. 2017. p. 142–77.

[86] King A. Diet in the Roman world: a regional inter-site comparison of the mammal bones. J Roman archaeol. 1999;12:168–202. doi: 10.1017/s1047759400017979

[87] Holmes M. Innovation, technology, and social change: The nature of the spread of the mouldboard plough and associated social consequences for human-animal relationships. In: McKerracher M, Hamerow H, editors. New perspectives on the medieval ‘agricultural revolution’. Liverpool: Liverpool University Press. 2022. p. 87–110.

[88] Holmes M, Thomas R, Hamerow H. Identifying draught cattle in the past: Lessons from large-scale analysis of archaeological datasets. Int J Paleopathol. 2021;33:258–69. doi: 10.1016/j.ijpp.2021.05.004 34044199

[89] Papworth M. The case for Chedworth Villa: exploring evidence for 5th-century occupation. Current Archaeol. 2021;373:18–25.

[90] Banham D, Faith R. Anglo-Saxon farms and farming. Oxford: Oxford University Press. 2014.

[91] Lewit T. Pigs, presses and pastoralism: farming in the fifth to sixth centuries AD. Early Medieval Europe. 2009;17(1):77–91. doi: 10.1111/j.1468-0254.2009.00245.x

[92] Moreland J. Land and Power from Roman Britain to Anglo-Saxon England?. Hist Mater. 2011;19(1):175–93. doi: 10.1163/156920611x564707

[93] Gerrard J. Rethinking the small pig horizon at York Minster. Oxon J Archaeol. 2007;26(3):303–7.

[94] Hammon A. Understanding the Romano-British–Early Medieval Transition: A Zooarchaeological Perspective from Wroxeter (Viroconium Cornoviorum). Britannia. 2011;42:275–305. doi: 10.1017/s0068113x11000055

[95] Loveluck C, Laing L. Britons and Anglo‐Saxons. The Oxford Handbook of Anglo-Saxon Archaeology. Oxford University Press. 2012. p. 534–55. doi: 10.1093/oxfordhb/9780199212149.013.0028

[96] Brandon P. The south downs. Cheltenham: The History Press. 2022.

[97] Holmes M. The ‘lamb of god’ in the early middle ages: A zooarchaeological perspective. J Mediev Hist. 2023;49(5):701–10.

[98] Moreland J. Concepts of the early medieval economy. In: Hansen I, Wickham C, editors. The long eighth century. Leiden: Brill. 2000. p. 69–104.

[99] Naylor J. Emporia and their hinterlands in the 7th to 9th-centuries AD: Some comments and observations from England. Rev Nord Archeol. 2016;24(2016):59–67.

[100] Pestell T, Ulmschneider K. Markets in early medieval Europe: Trading and ‘productive’ sites, 650-850. Macclesfield: Windgather Press. 2003.

[101] Holmes M. In: Lefèvre C, editor. Proceedings of the general session of the 11th international council for archaeozoology conference (Paris, 23-28 August 2010). Oxford: British Archaeological Reports: International Series. 2012. p. 57–68.

[102] Leahy K. Anglo‐Saxon Crafts. The Oxford Handbook of Anglo-Saxon Archaeology. Oxford University Press. 2012. p. 440–59. doi: 10.1093/oxfordhb/9780199212149.013.0024

[103] Rogers PW. From farm to town: The changing pattern of textile production in Anglo-Saxon England. Fasc Archaeol Hist. 2018;31:103–14.

[104] Whitelock D. English historical documents c.500-1042 volume 1. London: Routledge. 1996.

[105] Trow-Smith R. A history of British livestock husbandry to 1700. London: Routledge and Kegan Paul. 1957.

[106] Carroll CW, Wilson LH. The medieval shepherd: Jean de brie’s le bon berger (1379). Tempe (AZ): Arizona Center for Medieval and Renaissance Studies. 2012.

[107] Astill G, Grant A. The countryside of medieval England. Oxford: Blackwell. 1988.

[108] Faith R. The structure of the market for wool in early medieval Lincolnshire. Econ Hist Rev. 2012;65(2):674–700.

[109] Ryder M. Sheep and man. London: Duckworth. 1983.

[110] Blair J. Building Anglo-Saxon England. Oxford: Princeton University Press; 2018.

[111] Rose S. The wealth of England: The medieval wool trade and its political importance 1100–1600. Oxford: Oxbow Books. 2017.

[112] Swanton M. Anglo-Saxon prose. London: J.M. Dent. 1993.

[113] Holmes M, Hamerow H. The animal economy: A zooarchaeological perspective on agriculture and trade in Anglo-Saxon England. In: Hoggett R, Faulkner N, editors. The Anglo-Saxon agricultural revolution in Norfolk. Bicester: Archaeopress. 2025. p. 151–60.

[114] Oosthuizen S. Anglo-Saxon fields. In: Hamerow H, Hinton D, Crawford S, editors. The Oxford handbook of Anglo-Saxon archaeology. Oxford: Oxford University Press. 2011. p. 377–401.

[115] Halstead P. Calf mortality and milking: Was Tony Legge right after all?. In: Rowley-Conwy P, Serjeantson D, Halstead P, editors. Oxford: Oxbow. 2017. p. 119–25.

[116] Langdon J. Horses, oxen and technological innovation: The use of draught animals in English farming from 1066 to 1500. Cambridge: Cambridge University Press. 1986.

[117] Lloyd T. The English wool trade in the middle ages. Cambridge: Cambridge University Press. 1977.

[118] Holmes M. Foodways: From country to town. In: Christie N, Creighton O, editors. Transforming townscapes from burh to borough: The archaeology of Wallingford, AD 800-1400. 35. London: Society for Medieval Archaeology Monograph. 2013. p. 365–74.

[119] McKerracher M, Hamerow H. New perspectives on the medieval ‘agricultural revolution’. Liverpool: Liverpool University Press. 2022.

[120] Holmes M, Thomas R. …in winter, plough: Zooarchaeological evidence for the changing role of draught cattle and horses in medieval England AD 400-1400. In: Kropp C, Zoll L, editors. Draft animals in the past, present and future. Heidelberg: Propylaeum. 2022. p. 63–70.

